# An Integrative Strategy Delineates Modular Metabolic Remodeling and Potential Therapeutic Targets Across Metabolic Diseases

**DOI:** 10.1002/advs.76250

**Published:** 2026-06-22

**Authors:** Kuan Yang, Congxue Hu, Hang Zhao, Xiao Peng, Yiming Yan, Jiasi Wang, Wenqi Jiang, Depeng Mu, Yan Cao, Xia Li, Yunpeng Zhang

**Affiliations:** ^1^ Department of Urology in Cancer Hospital, College of Bioinformatics Science and Technology Harbin Medical University Harbin China; ^2^ School of Intelligent Medicine and Technology, Big Data Research Center Hainan Medical University Haikou China

**Keywords:** ligand–receptor signaling, metabolic diseases, metabolic module remodeling, single‐cell atlas, therapeutic targets, transcriptional regulation

## Abstract

Metabolic diseases are a substantial global health burden. However, how different metabolic pathways are connected to the clinical relevance in metabolic diseases remains unclear. Here, by integrating previously published single‐cell RNA sequencing datasets, a single‐cell metabolic atlas including 562 699 cells from 86 patients across seven major metabolic diseases is compiled. An advanced integrative analytical strategy to assess the activity of 227 metabolic pathways is developed. It is identified 89 disease‐enriched pathways. These pathways are further organized into 15 cooperative metabolic modules, revealing metabolic module remodeling associated with coordinated pathway activities and underlying regulatory mechanisms. By linking module activities to transcription factor dynamics and ligand–receptor signaling, cross‐cell‐type regulatory circuits underlying module‐specific remodeling and the identification of 63 potential therapeutic targets are delineated. Furthermore, an open‐access resource is developed to enable in‐depth exploration of the results in this study. In summary, this study illustrates conserved principles of modular metabolic remodeling across metabolic diseases and sheds light on therapeutic target discovery.

## Graphical abstract

An integrative single‐cell atlas across multiple metabolic diseases reveals coordinated metabolic modules and disease‐shared versus disease‐specific pathway activities. By systematically comparing scoring strategies, a robust RankAve framework is established. Coupled with network analysis and drug‐target prediction, this resource uncovers cross‐disease metabolic principles and provides a foundation for hypothesis‐driven therapeutic exploration.

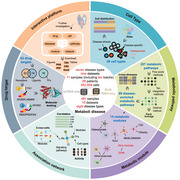

**Abbreviations**
GDM: gestational diabetes mellitus
L–R: ligand–receptor
MASLD/MASH: metabolic dysfunction‐associated steatotic liver disease/metabolic dysfunction‐associated steatohepatitis
MDs: metabolic diseases
MMs: metabolic modules
MPs: metabolic pathways
T2D: type 2 diabetes
TFs: transcription factors

## 1 Introduction

The prevalence of metabolic diseases (MDs), including obesity, type 2 diabetes (T2D), and hypertension, has increased considerably in recent decades, posing a substantial global health burden [1, 2, 3]. These disorders arise from disrupted metabolic processes and are characterized by complex, heterogeneous pathogenesis driven by the dysregulation of multiple interconnected metabolic pathways (MPs) [4].

Recent advances in single‐cell transcriptomics have enabled detailed investigations into the cellular and molecular mechanisms underlying metabolic dysfunction. Single‐cell studies have begun to elucidate how specific pathways contribute to disease progression [5]. For example, the activation of lipid metabolic programs in cardiomyocytes, including the ANGPTL1/VEGFR2 axis, promotes fibrosis in diabetic cardiomyopathy [6]. In osteoarthritis, AMP‐related genes such as PTGS2 and CYP2U1 exacerbate inflammation by modulating macrophage responses in the synovium [7]. However, most existing studies focus on isolated pathways. In contrast, aberrant metabolic activities typically result from the coordinated action of multiple pathways, yet the broader organization of this coordination remains poorly understood. The concept of metabolic modules (MMs), defined as groups of coregulated pathways that collectively influence disease phenotypes, has received limited attention, particularly at single‐cell resolution, where cellular heterogeneity and context‐specific metabolic reprogramming can be precisely assessed.

To address this gap, we developed a comprehensive single‐cell gene set scoring framework to systematically quantify metabolic activity across seven major MDs. By integrating multiple high‐quality single‐cell RNA sequencing (scRNA‐seq) datasets, we constructed a unified metabolic activity landscape that facilitates cross‐disease comparisons at the cellular, pathway, and module levels. An ensemble scoring strategy combined with network‐based analyses identified key MMs exhibiting disease‐specific activation patterns and characterized the transcription factors (TFs) regulating these programs. Further integration of transcriptional regulatory networks with ligand–receptor (L–R) interactions revealed intercellular communication axes coordinating metabolic states across cell types. Additionally, we developed an interactive Shiny platform to support intuitive exploration of these multilayered regulatory relationships. By integrating drug‐target data from the ChEMBL database, we demonstrate the utility of this atlas for drug target prioritization. Overall, this study provides the first system‐level atlas of metabolic regulation in MDs at single‐cell resolution and introduces a modular analytical framework to inform mechanistic studies and guide therapeutic target discovery.

## 2 Methods

### 2.1 ScRNA‐seq Data Collection, Integration, and Cell Type Annotation

It was curated publicly available scRNA‐seq datasets encompassing seven MDs from nine independent studies deposited in the Gene Expression Omnibus (GEO) database (Table S1). The raw count matrices were processed via the Seurat package (v.4.4.1/v.5.1.0). During the initial Seurat object creation, genes expressed in fewer than three cells (min.cells = 3) were removed. Low‐quality cells were subsequently excluded by filtering out barcodes with fewer than 200 detected genes (min.features = 200) and a mitochondrial gene expression ratio greater than 25%. No further global regression variables (e.g., vars.to.regress) were applied during the data scaling step to preserve biologically meaningful variations. Doublets were identified and removed via the scDblFinder R package (v.1.18.0). Following this filtering process, a total of 562 699 high‐quality cells, encompassing 39 151 genes across 71 samples (including ten batches), were retained to create a single‐cell transcriptomic meta‐analysis for subsequent analyses. After quality control, the raw counts were normalized via the NormalizeData function in Seurat. Highly variable genes (HVGs) were identified via the FindVariableFeatures function, and the top 2000 genes that consistently exhibited variability across datasets were selected, as supported by the HVG variance plot (Figure S1C,D). To prevent clustering from being disproportionately driven by cellular stress or technical factors, mitochondrial, ribosomal, and mitochondrial ribosomal genes were excluded from this HVG selection. Importantly, we emphasize that this exclusion was strictly limited to the dimensionality reduction and clustering steps. All mitochondrial and ribosomal genes were fully retained in the normalized expression matrix and were included in all downstream functional analyses, including the calculation of MP activity scores. Samples containing fewer than 200 cells and datasets with fewer than three samples were excluded before integration.

To mitigate interstudy batch effects while preserving biological variance, we applied the Harmony algorithm, based on the R package harmony (v.1.2.0), for cross‐dataset integration. Harmony has been shown to be robust for integrating datasets with highly unbalanced cell numbers across conditions (disease vs. control) and batches, as it iteratively corrects the principal component analysis (PCA) embeddings without forcing uniform cluster sizes. Importantly, we emphasize that differences in cell numbers between disease and control groups do not directly imply biological dominance and were therefore interpreted cautiously in all downstream analyses. PCA was performed on the HVG matrix, and the top 50 principal components were used as inputs for Harmony integration via RunHarmony function with default parameters. An elbow plot of the first 100 PCs supported the selection of 50 PCs, which captured the vast majority of biological variance (Figure S1E). Harmony integration was executed using the “sample” identifier as the batch variable to correct for technical variations arising from independent sample processing runs. The Harmony algorithm formally converged after 5 iterations. Unsupervised clustering and dimensionality reduction were subsequently performed. An elbow plot of the Harmony embeddings indicated that the variance explained plateaued around the 30th component (Figure S1F). Therefore, the first 30 Harmony components were used to construct a shared nearest neighbor (SNN) graph with FindNeighbors, followed by unsupervised clustering via FindClusters (resolution = 0.5). Dimensionality reduction and visualization were performed via RunUMAP (parameters: umap.method = “umap‐learn”, dims = 1:30, reduction = “harmony”). Clusters showing mixed marker expression from multiple major lineages were removed, and the integration and clustering steps were repeated to eliminate potential doublets. Cluster‐specific marker genes were identified via FindAllMarkers function, and clusters were annotated on the basis of canonical lineage markers to define major cell types. The resulting Harmony‐corrected embedding effectively mitigated batch effects across studies and disease types, ensuring that downstream analyses, including pathway scoring, TF inference, and cell‒cell communication modeling, reflected genuine biological heterogeneity rather than technical artifacts. To quantitatively assess the efficiency of data integration, the Local Inverse Simpson's Index (LISI) was calculated using the R package lisi (v.1.0). The integration LISI (iLISI) was computed to evaluate the mixing of technical batches (sample and patient) and biological conditions (disease), while the cell‐type LISI (cLISI) was used to assess the preservation of cell‐type purity.

To further preserve the cell‐type annotations that were partially lost during joint integration and ensure the highest fidelity to the original published findings, we implemented a reference‐based label‐transfer strategy via Seurat. The nine individual datasets were merged into a single reference object via unique cell identifiers. Transfer anchors between the reference dataset and each query dataset were identified via FindTransferAnchors function with the first 30 principal components. The cell‐type labels from the reference dataset were mapped to the query datasets via TransferData function, and the predicted labels, along with their scores, were added to the query metadata via AddMetaData function. Cells with maximum prediction scores below a defined threshold (for example, 0.6) were labeled ambiguous. Original annotations from the query datasets were retained for comparison, allowing evaluation of mapping quality and concordance. Prediction performance was assessed via bubble plots, histograms of prediction scores, and purity plots showing the maximum cluster proportion for each cell type. Reference‐based annotations were further validated via violin plots and feature plots of canonical marker genes. The final annotated datasets, including both mapped labels and original metadata, were saved for downstream analyses.

### 2.2 Tissue Distribution Preference Analysis

For tissue enrichment analysis, we employed the Ro/e method as detailed by Zhang et al. The Ro/e index was calculated via the calTissueDist function in the R package STARTRAC (v.0.1.0). The expected cell numbers for each cell subpopulation or cellular program in combination with the tissue type were derived via the χ^2^ test (method = ‘chisq’). The Ro/e value > 1 indicates enrichment of a specific cell subpopulation or cellular program in a particular tissue type.

### 2.3 Evaluation and Optimization of MP Integration Strategies

To achieve robust and unified representations of pathway activity across single cells, we systematically compared and optimized integration strategies that combine five widely used single‐cell scoring methods: AddModuleScore [8], AUCell [9], UCell [10], GSVA [11], and ssGSEA [12]. We extracted 227 metabolism‐centered MPs from four major pathway databases, KEGG, Reactome, Hallmark, and WikiPathways, together with relevant published studies [13, 14, 15, 16, 17, 18] (Table S4). To ensure consistency and reduce redundancy across sources, pathways with overlapping or equivalent biological definitions were manually harmonized and assigned unified names. For each consolidated pathway, gene sets from different databases were merged, and duplicate genes were removed to generate a non‐redundant union gene set. To facilitate community access, the complete list of curated MPs and all associated supplementary data tables are freely accessible and can be retrieved from the ‘Download’ page of our interactive online platform (https://scmetabolismexplorer.shinyapps.io/shiny/). For each of the 227 curated MPs, pathway activity matrices were constructed, with each row representing a single cell and each column corresponding to one of the five scoring methods. We developed ten mathematically defined integration strategies on the basis of distinct statistical principles: MeanScore (arithmetic mean of the five scores), MedianScore (median value, robust to outliers), RankAve [19] (average normalized rank across scoring methods), HarmonicMean [20] (harmonic mean emphasizing concordantly high scores), TrimmedMean [21] (mean after removing the upper and lower 10% of values), WeightedMean (variance‐weighted mean using method‐specific variances as weights), RankProduct [22] (geometric mean of ranks capturing consistent rank shifts), MaxScore (maximum of the five scores), MinScore (minimum of the five scores), and ZSum [23] (sum of z‐transformed values after standardizing each scoring method across cells). Each strategy aggregated the outputs of the five individual scoring methods to generate an integrated pathway activity score for every cell. These ten integration strategies represent distinct aggregation principles, including direct combination of raw scores, rank‐based integration, extreme‐value extraction, and standardized‐score fusion. Accordingly, they differ in their sensitivity to scale heterogeneity, outliers, concordance across methods, and preservation of relative pathway ordering. To improve computational efficiency, all the integration procedures and performance evaluations were parallelized via the R packages doParallel (v.1.0.17) and foreach (v.1.5.2).

The performance of each integration strategy was systematically assessed across three complementary dimensions: 1) Correlation consistency: For each pathway, Spearman's rank correlation coefficients were calculated between each integration strategy and the five original scoring methods. The mean correlation across methods was used to quantify overall agreement. The distributions of the mean correlations across all pathways were visualized via combined violin–box plots, and the global mean correlation (GM*m*) for each strategy was recorded. 2) Robustness through random resampling: To evaluate robustness, ten independent random samplings were performed, each retaining 5% of the cells per pathway. For each iteration, correlations were recomputed, and the mean correlation across ten repetitions (TenBM*m*) reflected the reproducibility of the integration outcomes under stochastic sampling. 3) Stability via bootstrap resampling: To measure stability, we conducted 100 bootstrap iterations by sampling 80% of the cells with replacement. Spearman correlations between bootstrapped and complete datasets were calculated, and the standard deviation of these correlations (SD*m*) was used as an inverse stability index, with a lower SD indicating greater stability. Finally, a composite performance score was computed to integrate these three metrics as follows:

$$Finalm=GMm+TenBMm\;-\;\left(SDm\right)\;\left(m=1,\;2,\;\text{…},\;10\right)$$

This unified score rewards methods with higher correlation consistency and robustness while penalizing instability. Comparative visualization via R package ggplot2 (v.3.5.1) revealed that RankAve, the average of normalized ranks, achieved the highest composite score, indicating the most stable and consistent integration strategy for MP activity across single cells. This performance may be attributed to the ability of RankAve to minimize the influence of score‐scale differences and extreme values across methods, while preserving the relative ordering of pathway activity across cells. As such, RankAve provides a balance between cross‐method consistency, robustness, and stability. To further validate the generalizability of the framework, we applied the same integration and evaluation procedures to bulk RNA‐seq datasets obtained from public databases, whose expression matrices were uniformly converted into transcripts per million (TPM) format for subsequent pathway activity scoring. These bulk data were used as an additional validation layer to assess the performance of the framework across independent transcriptomic datasets and different tissue backgrounds. The results suggested that RankAve demonstrated more consistent performance than other methods, supporting its robustness across both single‐cell and bulk transcriptomic contexts.

### 2.4 Identification of MMs via WGCNA

To explore the coordinated regulation of disease‐associated MPs, we performed WGCNA using the R package WGCNA (v.1.73) on the 89 pathways identified as significantly enriched in the disease group. Single‐cell pathway activity scores were aggregated at the patient level to generate a pseudobulk pathway–patient matrix. For each patient, the mean pathway activity across all cells was calculated, yielding a matrix with patients as rows and pathways as columns. While high‐dimensional WGCNA (hdWGCNA) is highly effective for identifying intra‐cellular gene co‐expression networks, we specifically applied WGCNA in a pseudobulk patient‐versus‐pathway context. This strategic design allowed us to capture systemic, macro‐level pathway coordination across different patients, directly linking metabolic modules to clinical disease status, heterogeneity, and comorbidity. Before network construction, the dataset was filtered via the goodSamplesGenes function in WGCNA to remove pathways or patients with excessive missing values or low variance. An appropriate soft‐thresholding power was selected via pickSoftThreshold function to ensure approximate scale‐free topology, typically choosing the smallest power at which the scale‐free topology fit index R^2^ approached 0.9.

The adjacency matrix was computed via the selected soft‐thresholding power with Pearson correlation to quantify linear relationships in metabolic pathway activities across patients. Given the non‐standard application of WGCNA to pathway‐level data, we evaluated the sensitivity of module detection to the choice of correlation metric. Specifically, we repeated the analysis using Spearman correlation and compared the resulting module assignments with those obtained using Pearson correlation. The adjusted Rand index (ARI), calculated using the R package mclust (v.6.1.1), was 0.559, indicating a moderate level of concordance between the resulting module structures [24, 25]. We further summarized the correspondence between the two analyses in Table S6, which provides a detailed comparison of module assignments and eigengene correlations between the Pearson‐ and Spearman‐based results. This was followed by calculation of the topological overlap matrix (TOM) to measure pathway interconnectedness. Hierarchical clustering of pathways on the basis of TOM dissimilarity was performed via average linkage, and modules of coexpressed pathways were identified via the cutreeDynamic function, with a minimum module size of three pathways. Module assignments were converted to distinct colors for visualization. This procedure yielded 15 MMs, each representing a set of pathways with coordinated activity across patients. The clustering dendrogram and module colors were visualized via plotDendroAndColors function, and module assignments for all pathways were saved for downstream analyses of disease specificity, TF associations, and cell–type–level regulation.

### 2.5 Transcriptional Activity Analysis

TF activity was determined via the R package decoupleR (v.2.12.0). The network from OmniPath, obtained for ‘human’ without splitting complexes, was filtered to include TFs detected in both the control and disease groups, identifying 752 testable TFs. Using the Wilcoxon test, we identified 491 TFs that were highly expressed in the disease groups (*p* < 0.05). Subsequently, pseudobulk analysis was performed via the R package limma (v.3.62.2) to further refine the set to 479 TFs at the patient level (single disease versus other diseases; screening criteria: *p* value < 0.05 and log fold‐change > 0.25).

### 2.6 Cell–Cell Communication Analysis

Cell–cell communication was investigated via the R package CellChat (v.1.6.1) to infer molecular interaction networks between different cell types across cellular programs. L–R pairs with *p* < 0.05 were considered significant interactions between different cell types in different diseases.

### 2.7 Shiny Web Application Development

The Shiny application (termed scMetabolismExplorer) was developed in R via the Shiny framework (v.1.9.1). This application enables visualization of study results and the download of relevant data, allowing researchers to access datasets and the analytical outcomes of interest directly. Refer to Figure 7 for an overview.

### 2.8 Drug Screening and Molecular Docking of Core Targets

We used the Python package Drug2cell [26] (v.0.1.2) to screen for potential drug targets and targeted drugs for L–R pairs and TFs. SDF files of potential drugs and 3D structural models of core target proteins were sourced from PubChem and the PDB database (https://www.rcsb.org/). CB‐Dock2 was used to preprocess small molecule ligands and target proteins, including energy optimization, hydrogenation, water molecule removal, and energy minimization [27]. The docking site with the lowest Vina score was selected as the optimal binding mode. The three‐dimensional and two‐dimensional structures of the ligand‒receptor complex were generated via Discovery Studio 2025 software (https://www.3ds.com/products/biovia/discovery‐studio).

### 2.9 Statistical Analysis

Statistical analyses were performed via R (v.4.4.1). Two‐group comparisons were performed using Wilcoxon tests, whereas comparisons across multiple groups were performed using ANOVA. Benjamini–Hochberg (BH)‐adjusted *p* values < 0.05 were considered statistically significant.

## 3 Results

### 3.1 A Single‐Cell Atlas of human Metabolic Diseases

**FIGURE 1:**
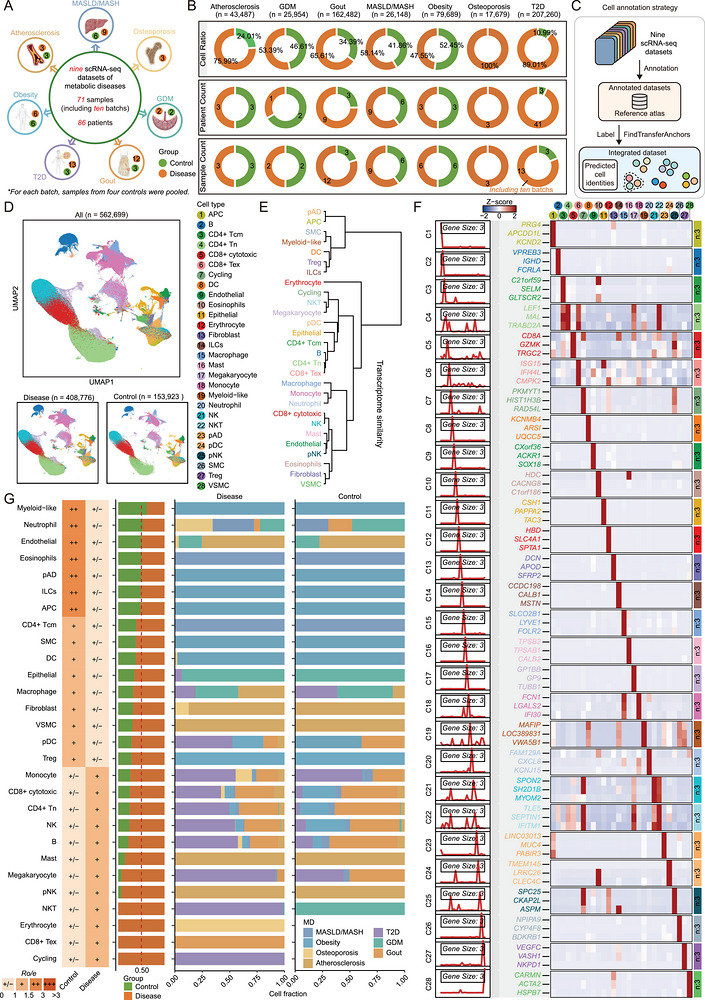
Overview of the human MD single‐cell atlas. (A) Summary of all datasets incorporated in this study. (B) Overview of MD datasets, including the number of samples, number of patients, and proportion of cells. (C) Schematic of cell annotation. (D) UMAP plot showing overall cell types (top) and their tissue origins (bottom). (E) Hierarchical clustering of cell types on the basis of global transcriptional similarity. (F) Heatmaps displaying the top three marker genes in 28 cell types. (G) Tissue preference of the 28 cell types in the disease and control groups, as revealed by the Ro/e (ratio of the observed cell number to the expected cell number) (left). Stacked bar plot showing the distribution of the 28 cell types in different groups (middle) and MDs (right).

To generate a comprehensive single‐cell atlas of human metabolic diseases (MDs), we systematically integrated published scRNA‐seq datasets from control and disease tissues across seven pathological conditions. The final cohort comprised 71 samples from 86 patients, encompassing atherosclerosis, gestational diabetes mellitus (GDM), gout, metabolic dysfunction‐associated steatotic liver disease/metabolic dysfunction‐associated steatohepatitis (MASLD/MASH), obesity, osteoporosis, and T2D (Figure 1A,B and Table S1). The robustness of the large‐scale integration was validated through multiple complementary assessments. Quality control metrics were comparable across datasets, with no evident technical bias (Figure S1A, B). Integration parameters were optimized based on variance diagnostics, including the selection of 2000 highly variable genes and appropriate dimensionality (PC = 50; Harmony = 30) (Figure S1C–F). Quantitative evaluation using LISI demonstrated that Harmony effectively reduced batch effects, as indicated by increased iLISI scores for ‘Sample’ and ‘Patient’ compared to PCA, while preserving cellular identity, with cLISI values remaining close to 1.0. Notably, low iLISI scores for ‘Disease Mixing’ further indicated that disease‐specific cellular heterogeneity was retained rather than confounded by technical variation (Figure S1G). Following stringent quality control, cell types were annotated via canonical lineage markers. To recover the cellular identities potentially obscured during integration, a reference‐based label‐transfer approach was applied, in which reference annotations were projected onto query datasets. This enabled the classification of 562 699 cells into 28 distinct cell types (Figure 1C,D; Figure S2A; see Methods). Annotation accuracy was confirmed through prediction scores and cluster purity assessments, with most cell types exhibiting high confidence in their assigned identities (Figure S2D). Each annotated population predominantly localized to a single cluster with minimal transcriptional overlap, supporting the robustness of the classification framework (Figure S2E). Collectively, these results demonstrate that our annotation strategy allows precise and reproducible identification of major cellular constituents.

The transcriptional relationships among the 28 cell types were further analyzed, revealing patterns of relatedness consistent with the known spatial and functional organization of tissues [28] (Figure 1E). The marker gene profiles revealed distinct molecular signatures for each cell type (Figure 1F and Table S2). Comparative analysis of cell type distributions across control and disease conditions revealed substantial alterations in cellular composition (Figure 1G; Figure S2F,G). Notably, myeloid‐like cells, preadipocytes (pADs), innate lymphoid cells (ILCs), adipocyte precursor cells (APCs), smooth muscle cells (SMCs), and regulatory T (Treg) cells are uniquely observed in obesity. Eosinophils and CD4^+^ central memory T cells (CD4^+^ Tcm) are restricted to MASLD/MASH, whereas vascular smooth muscle cells (VSMCs), mast cells, and proliferating natural killer (pNK) cells are predominantly associated with atherosclerosis. Furthermore, to delineate these enrichment patterns across biological contexts, we assessed the tissue‐specific distribution of each cell type based on their sampling origins (Figure S2B,C). This analysis uncovered distinct and biologically coherent microenvironmental signatures. Eosinophils and CD4^+^ Tcm cells were predominantly enriched in white blood cells (WBC), whereas multiple stromal and immune populations exhibited strong tissue restriction. Notably, pADs, APCs, and ILCs preferentially accumulated in white adipose tissue (WAT), while VSMCs, fibroblasts, and mast cells were specifically enriched within atherosclerotic plaques. These findings indicate that MDs arising from distinct tissue origins are driven by specific, context‐dependent cellular populations, underscoring the presence of tissue‐specialized regulatory mechanisms that shape disease pathogenesis.

Among the shared cell types, transcriptionally similar subsets presented consistent tissue distributions. In T2D, CD8^+^ cytotoxic T cells and NK cells are enriched in disease samples, reflecting their transcriptomic proximity and corroborating prior evidence of increased cytotoxicity and clonal T‐cell expansion [29]. The number of plasmacytoid dendritic cells (pDCs) is markedly elevated in T2D, which is consistent with their reported role in vascular dysfunction [30]. Macrophages are significantly enriched in GDM, which is in line with their contribution to dysregulated placental immunity, where inflammatory macrophages, dendritic cells, and Th1 cells promote insulin resistance and GDM pathogenesis [31]. Endothelial cells were preferentially enriched in control tissues, indicating a pronounced shift in tissue composition and the microenvironment between healthy and diseased states. Similar patterns were observed in women with a history of GDM, which is consistent with the notion that early endothelial dysfunction may precede cardiometabolic disorders [32]. Overall, these findings reveal progressive, disease‐specific remodeling of the immune microenvironment across MDs. The selective accumulation of cytotoxic lymphocytes, together with altered endothelial representation, suggests a transition from homeostasis to an activated, dysregulated immune state during MD progression. This immunological reconfiguration likely underlies chronic inflammation, tissue damage, and systemic metabolic deterioration.

### 3.2 An Integrative Analytical Strategy Reveals Disease‐Enriched Pathways

**FIGURE 2:**
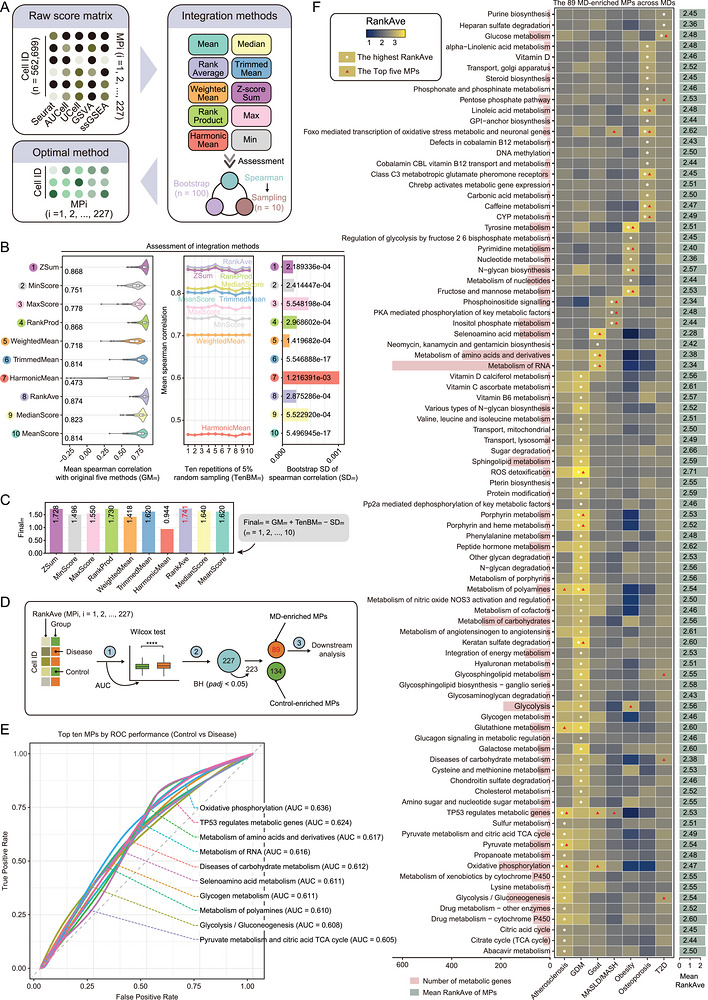
Development of a single‐cell metabolic scoring framework and detection of MD‐enriched MPs. (A) Overview of the metabolic scoring framework. (B) Average correlations between the ten integration methods and the original scoring approaches (left), mean correlations over ten iterations (middle), and overall stability (right). (C) Comparison of the aggregated performance scores across the ten integration methods. (D) Workflow for identifying pathways enriched in MDs via the RankAve approach. Differential pathways were defined via Wilcoxon tests with adjusted *p* < 0.05. (E) ROC curves for the top ten MPs ranked by AUC. (F) Number of metabolic genes in each MP (left), enrichment landscape of 89 MPs across diseases (middle; white dots denote MPs with the highest RankAve scores; red triangles denote the top five MPs ranked by RankAve scores in MDs), and average RankAve scores for each MP across diseases (right).

Given the crucial role of metabolic reprogramming in the onset and progression of MDs, we developed a single‐cell metabolic scoring framework to quantify pathway‐level alterations. Pathway activity scores were computed for each cell via five independent gene‐set scoring approaches, and ten integration strategies were systematically evaluated. RankAve showed favorable performance and was selected as the integrative approach, demonstrating superior concordance with individual scoring methods and robust performance across resampling and bootstrap analyses (Figure 2A,B; see Methods). It also showed more consistent performance than other strategies in overall scoring accuracy at both the single‐cell and bulk transcriptomic levels (Figure 2C; Figure S3A).

Utilizing RankAve‐integrated scores, we constructed a cell‐by‐pathway matrix and identified 223 metabolic pathways (MPs) showing differential representation between disease and control samples. Among these, 89 MPs consistently exhibited disease‐associated enrichment and were chosen for downstream analysis (Figure 2D). Their discriminatory power was reinforced by receiver operating characteristic (ROC) analyses, with the top‐ranked pathways achieving clear separation between disease and control samples across both single‐cell and bulk datasets (Figure 2E; Figure S3B,C). An examination of pathway enrichment across individual MDs revealed disease‐specific metabolic signatures (Figure 2F). In atherosclerosis, enriched pathways were predominantly related to energy metabolism remodeling and cytochrome P450 (CYP450)‐mediated drug and xenobiotic metabolism, processes previously implicated in disease pathogenesis [33, 34]. In GDM, enriched pathways involved carbohydrate, lipid, and amino acid metabolism, as well as oxidative stress regulation, which is consistent with known hallmarks of insulin resistance, lipid dysregulation, and oxidative damage [35].

To highlight the dominant metabolic features within each MD, the top‐ranked pathway was identified for each condition. In atherosclerosis, glutathione metabolism is most prominent, underscoring the role of redox homeostasis in vascular inflammation and plaque progression [36]. In GDM, reactive oxygen species (ROS) detoxification has emerged as the leading pathway, reflecting oxidative stress‐driven insulin resistance and placental dysfunction [37]. Gout is characterized by selenoamino acid metabolism, indicating altered amino acid‐related metabolic patterns. In MASLD/MASH, phosphoinositide signaling was predominant, highlighting lipid‐related signaling in hepatic metabolic regulation. Obesity is associated with increased tyrosine metabolism, suggesting that altered amino acid metabolism is associated with systemic energy imbalance [38]. Osteoporosis is linked primarily to class C3 metabotropic glutamate receptor‐related pathways, suggesting a potential connection between amino acid‐related signaling and bone metabolism [39]. Finally, glucose metabolism ranked highest in T2D patients, directly reflecting the central disruption of glucose homeostasis underlying disease pathophysiology [40]. Collectively, these findings demonstrate that the 89 MD‐enriched pathways capture disease‐specific metabolic perturbations and provide a framework for identifying key metabolic processes that may serve as potential therapeutic targets.

### 3.3 Modular Organization of Metabolic Pathways and Altered Activity States in Metabolic Diseases

**FIGURE 3:**
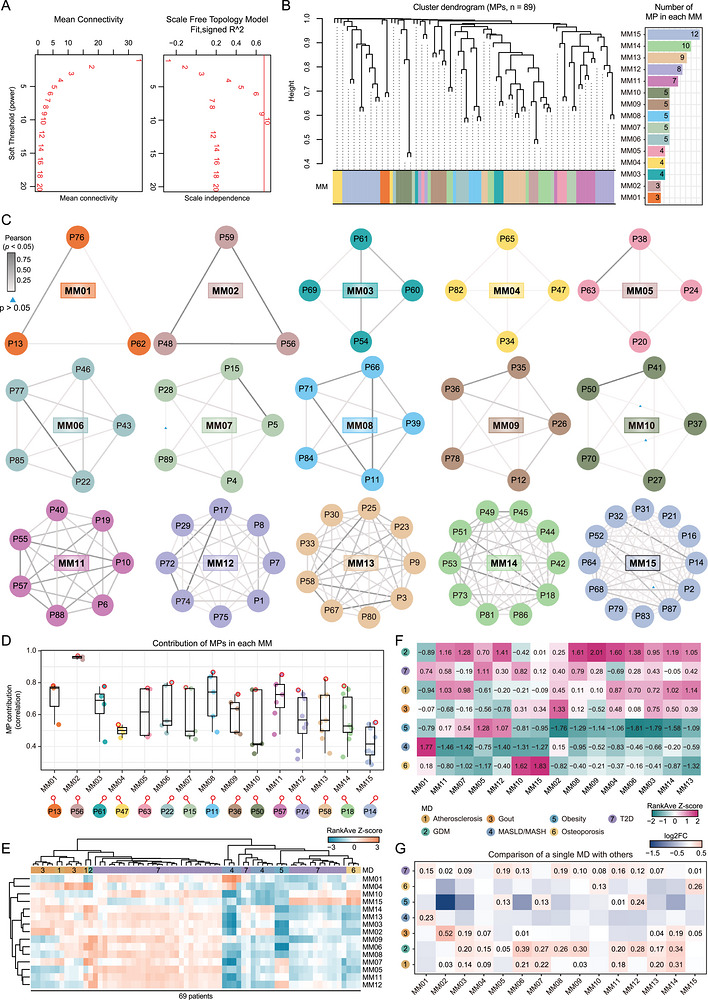
Identification of MMs associated with multiple MPs via WGCNA. (A) Evaluation of scale‐free topology across soft‐thresholding powers, showing mean connectivity (left) and scale independence (right). (B) Dendrogram of co‐expression modules (left) and the number of MPs contained in each MM (right). (C) Network of pairwise Pearson correlations among MPs in each MM, with blue triangles indicating non‐significant associations (*p* > 0.05). (D) Box plots showing the MP with the strongest intramodule contribution on the basis of Pearson correlation coefficients in multiple MMs. (E) Heatmap of patient‐level MM scores estimated via RankAve, annotated by disease type. (F) Heatmap of disease‐level MM scores hierarchically clustered across MDs. (G) Log2 fold‐change heatmap showing the relative enrichment of each MM in a given MD compared with all others.

The pathogenesis and progression of MDs are driven by complex interactions among multiple MPs, leading to systemic dysregulation [41]. To characterize these patterns and potential coordinated interactions among enriched MPs, we applied the weighted gene co‐expression network analysis (WGCNA) to construct an MP co‐expression framework using pseudobulk transcriptomic data from 69 patients. A soft‐thresholding power of β = 10 was chosen according to the scale‐free topology criterion (Figure 3A). Topological overlap matrix analysis and hierarchical clustering identified 15 distinct metabolic modules (MMs), with robust intramodular correlations indicating coordinated metabolic alterations and stable module definitions (Figure 3B,C). The contribution of individual MPs to overall module expression varied, reflecting differences in internal coordination. MM01 is dominated by inositol phosphate metabolism, a pathway central to nutrient sensing and energy homeostasis [42], suggesting a nutrient‐responsive metabolic program. MM02 exhibited strong pathway coordination, with “metabolism of amino acids and derivatives” as its core pathway, regulating energy balance through mTOR and GCN2/ATF4/FGF21 signaling [43]. Selenoamino acid metabolism and RNA metabolism further positioned MM02 as an integrated metabolic–transcriptional regulatory unit [44]. MM15 encompassed the largest number of MPs, with linoleic acid metabolism as the top contributor, reflecting its role in excessive adipogenesis and inflammation [45], highlighting MM15 as a key functional module in lipid dysregulation (Figure 3D; Figure S4A).

Patient‐level RankAve scores were used to evaluate disease‐specific patterns across MMs. Patients with atherosclerosis, GDM, and gout displayed consistently elevated scores across multiple modules, indicating coordinated disturbances in several energy MPs (Figure 3E). In contrast, MASLD/MASH, obesity, and osteoporosis exhibited high scores in only a subset of modules. T2D showed marked heterogeneity, with some patients displaying activation across multiple MMs and others dominated by only a few, reflecting distinct metabolic adaptation strategies. Comparative analysis of module‐level scores and log2 fold‐changes delineated the MM preference landscape (Figure 3F,G; Figure S4B). GDM exhibited the most extensive module engagement, spanning nine MMs, notably MM04, MM08, and MM09, with coordinated activity among several modules, indicating broad and complex metabolic abnormalities (Figure S4D). MASLD/MASH and gout preferentially engaged MM01 and MM02, reflecting lipid‐ versus amino acid‐driven metabolic signatures. Atherosclerosis, obesity, and osteoporosis rely on a limited number of MMs, indicating focused disease‐specific metabolic alterations.

We further examined the relationships between MMs and disease‐associated cell types. Most MMs presented the highest scores in macrophages, epithelial cells, and cycling cells, which is consistent with their diverse metabolic demands and central roles in disease progression (Figures S4C and S5A). CD8^+^ cytotoxic T cells scored highest in MM02, particularly in gout, suggesting that amino acid metabolism and energy programs enhance T‐cell effector activity and contribute to inflammation (Figure S4A,C). In MASLD/MASH patients, MM01 scores were elevated in monocytes and neutrophils, suggesting that metabolic remodeling within these populations may shape the local immune landscape (Figure S5B,C and Table S5). Associations between MMs and immune pathway activity revealed module‐specific patterns (Figure S4E and Table S3). Several MMs, especially MM14, are strongly correlated with both antigen presentation and immune evasion pathways, indicating dual roles in immune activation and suppression. MM15 is weakly associated with most immune pathways but is positively correlated with cytokine and chemokine signaling, particularly in osteoporosis, reflecting potential effects on bone‐related cells [46]. In obesity, the chemokine, cytokine, TNF, and TGFβ signaling pathways are markedly enriched, with elevated TGFβ activity indicating ongoing adipose tissue fibrosis [47]. In atherosclerosis, TNF signaling is mainly mediated by MM11 and MM14, suggesting that specific MMs may drive inflammatory responses and influence endothelial function, monocyte recruitment, and plaque stability [48]. Collectively, these findings provide a systematic view of coordinated immune–metabolic dysregulation across MDs and highlight the potential of MMs as immune–metabolic regulatory targets.

### 3.4 Atlas of Metabolic Module–Transcription Factor Associations in Metabolic Diseases

**FIGURE 4:**
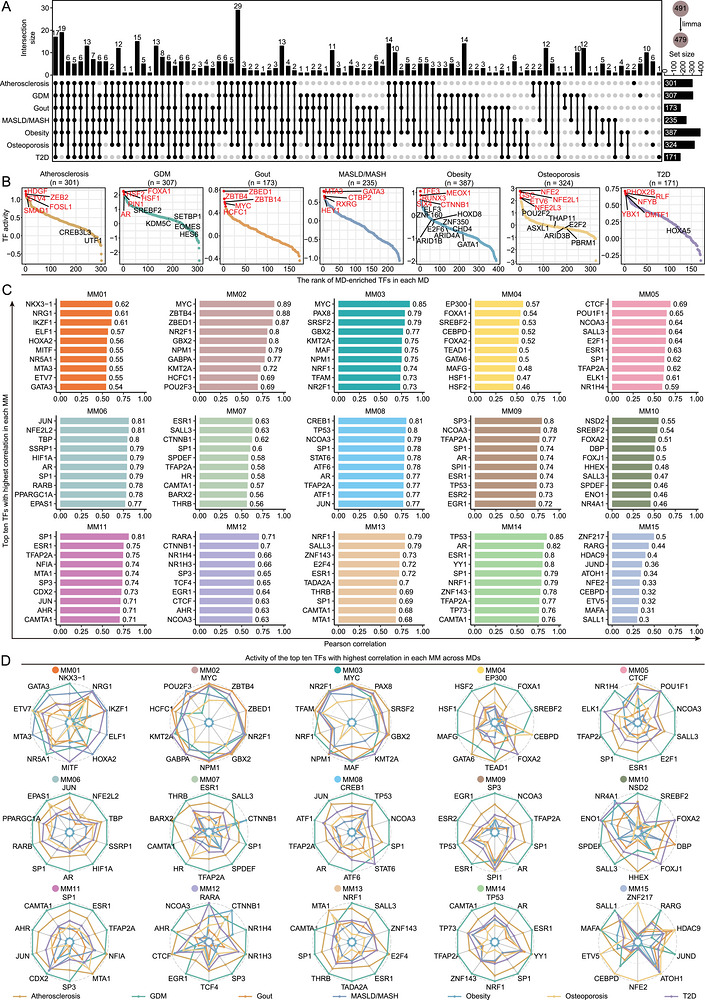
TF‒MM associations across MDs. (A) UpSet plot summarizing shared and disease‐specific TFs. (B) Rank‐ordered distribution of TFs enriched in each MD; points denote individual TFs, with red indicating the top five TFs per disease and black signifying TFs with disease‐specific expression patterns. (C) The top ten TFs most strongly correlated with each MM. (D) Radar plots showing the activity profiles of these TFs across MDs, represented by normalized expression levels.

Transcription factors (TFs) orchestrate gene expression programs by binding specific genomic sequences, playing a pivotal role in determining cellular identity and function [49]. We hypothesized that distinct upstream TFs regulate the previously identified MMs. To clarify their regulatory contributions, a systematic identification of MD‐associated TFs was performed. Initial screening yielded 751 candidate TFs across all samples, which were refined via statistical tests to 491 TFs with significant disease‐associated expression specificity. The aggregation of single‐cell profiles into pseudobulk matrices and additional filtering produced a high‐confidence set of 479 TFs for downstream analyses. The distribution of curated TFs across the seven MDs revealed substantial regulatory heterogeneity (Figure 4A,B). Most MDs were enriched for uniquely associated TFs; however, neither gout nor MASLD/MASH exhibited disease‐specific TFs, suggesting a more diffuse transcriptional landscape. In contrast, the remaining five MDs presented distinct TF signatures. Notably, a conserved core of 17 TFs was consistently present across all the MDs, indicating shared pathogenic mechanisms. These patterns parallel clinical epidemiology: MDs frequently cooccur and share mechanistic underpinnings. For example, MASLD/MASH progression is strongly linked to obesity, T2D, and insulin resistance, whereas MASLD/MASH patients have a 71% greater risk of developing gout [50, 51]. The high prevalence of diabetes and obesity among gout patients further supports shared transcriptional bases [52].

To functionally contextualize TFs, their associations with MMs were quantified, revealing pronounced heterogeneity indicative of both disease‐specific and shared regulatory logic (Figure 4C). In gout‐associated MM02, MYC, NPM1, and GABPA, established regulators of cell growth and proliferation [53, 54, 55, 56], showed strong positive correlations with module activity, suggesting a disease‐specific role. In atherosclerosis‐associated MM11, activity has been linked to NFIA, a regulator of fatty acid metabolism [57], and AHR, a mediator of exogenous and endogenous signaling [58]. MM11 was also enriched in GDM, indicating that these TFs may function as shared regulators connecting distinct diseases. Within osteoporosis‐associated MM15, several TFs have been identified, including ZNF217 and RARG (implicated in hematologic malignancies [59, 60, 61]), HDAC9 (linked to vascular inflammation), and JUND (activated in obesity [62, 63]), highlighting that molecular convergence may contribute to comorbidities (Figure 4D).

To determine the cellular context, the expression of the top ten TFs from disease‐associated MMs was examined across cell types (Figure S6A). In gout‐associated MM02, MYC, NPM1, and GABPA were predominantly expressed in B and T cells, suggesting roles in immune–metabolic regulation specific to gout. The coordinated co‐expression of these genes indicates integrated transcriptional control within immune populations (Figure S7A). In MM11 associated with atherosclerosis, NFIA and AHR are highly expressed in endothelial cells, fibroblasts, macrophages, and mast cells, indicating that they are involved in vascular remodeling and inflammation [64, 65, 66]. In GDM, NFIA and AHR exhibited high expression in endothelial cells and macrophages, respectively, demonstrating that identical TFs can mediate distinct MMs in different cellular contexts. Overall, TFs in MDs exhibit a dual regulatory architecture: they function in cell‐type‐specific manners while also acting as shared regulatory nodes across MDs. This core–shell organization underlies disease heterogeneity, comorbidity, and convergent pathophysiology, providing a mechanistic framework for transcriptional regulation in MDs.

### 3.5 Identifying the Key Associations Between Ligand‒Receptor Signaling and Transcription Factors in Metabolic Modules

To investigate shared regulatory characteristics among MDs, we systematically characterized intercellular communication networks across all cell types and identified significantly expressed ligand–receptor (L–R) pairs in key cells. To define the transcriptional circuits underlying these interactions, TF–L–R regulatory relationships were integrated to examine connections between upstream TFs and L–R pairs. Mechanistic mapping revealed a strong correspondence between cell types involved in major L–R interactions and those exhibiting high activity scores in representative MD‐related MMs (Figure 6A; Figures S5B and S8A–G). This concordance underscores the functional relevance of MMs in MD pathophysiology. Immune cells were consistently identified as central mediators of intercellular communication across all the MDs (Figures S8A–G and S9A–G), confirming their established roles in metabolic dysfunction. For example, macrophage subpopulations undergo dynamic changes during MASLD/MASH progression [67], T cells sustain chronic low‐grade inflammation in obesity through cytokine secretion [68], and B cells increase osteoclast activity in osteoporosis via the RANKL–OPG axis [69]. Collectively, these observations establish immune cells as key regulators of MD‐associated signaling networks.

**FIGURE 5:**
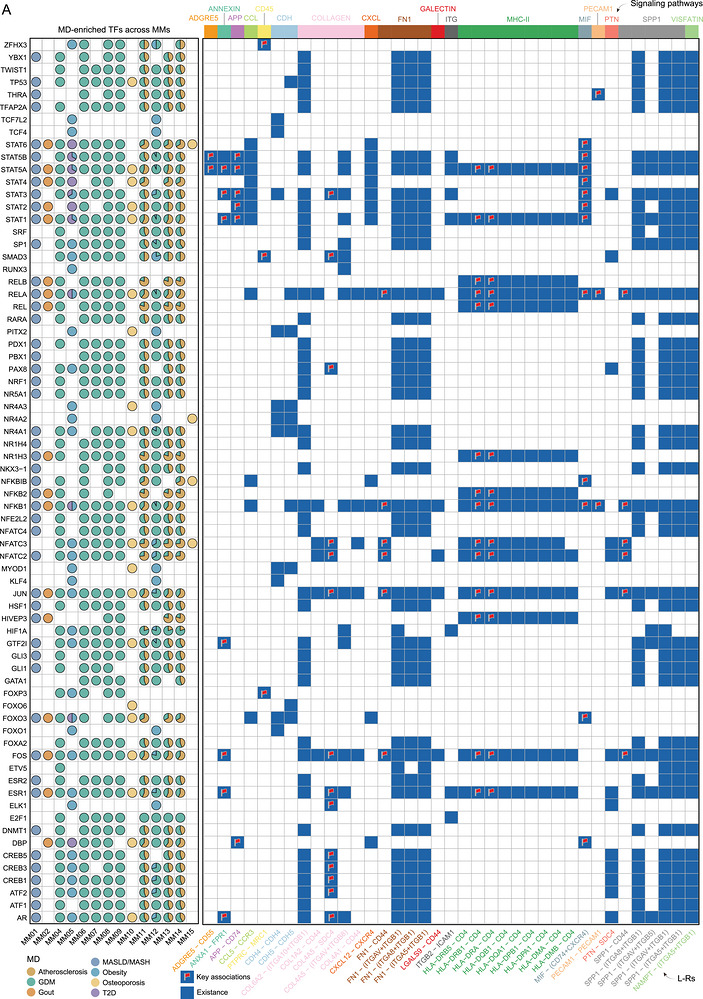
Regulatory relationships between TFs and L–R pairs. (A) Scatter plot illustrating the associations between TFs and MMs across MDs. The pie chart, color‐coded by disease type, displays the proportion of L‒R pairs regulated by TFs (left). Heatmap showing the regulatory interactions between TFs and L–R pairs, with the top annotation indicating the signaling pathways associated with each pair (right).

An integrated hierarchical framework was constructed by mapping TF–L–R relationships onto MMs. Members of the STAT family occupy central positions across multiple MMs (Figure 5A), reflecting broad functional roles. Upon stimulation, STAT1 mediates insulin signaling to the nucleus, whereas STAT3 is essential for immune regulation and bone formation [70, 71]. STAT5B, which is required for lymphocyte development and survival [72], also promotes angiogenesis in diabetes by supporting endothelial proliferation [73]. Although these TFs participate in diverse biological processes, their widespread presence across MMs complicates the attribution of specific functions to individual MDs or modules. STAT6 was an exception, showing marked enrichment in MM08 (Figure 4C). STAT6 regulates MMs through immune‐related signaling pathways, such as the CCL5–CCR3 axis (Figure 5A), and can be activated independently of canonical JAK‐mediated phosphorylation, potentially explaining its module‐specific effects. RELA and NFKB1 displayed regulatory patterns similar to those of the STAT family, with broad activity across modules without clear specificity.

In addition to these global regulators, several TFs, including ZFHX3, TCF4, KLF4, FOXP3, and E2F1, were identified as regulators of specific L–R pairs involved in disease progression. TCF4, a key regulator of adipocyte metabolism [74], coregulates MM05 and MM12 with KLF4. Loss of TCF4 in high‐fat diet models impairs insulin sensitivity, increases body weight, and expands subcutaneous adipose tissue, increasing susceptibility to diabetes. E2F1, which is primarily known for its ability to regulate the cell cycle, also contributes to the development of metabolic tissues, including the pancreas, adipose, muscle, and liver, by influencing adipogenesis, glucose homeostasis, and muscle physiology [75]. Together with FOXP3 and ZFHX3, E2F1 regulates key modules associated with GDM. Overall, these analyses establish a cross‐cell‐type signaling network that integrates transcriptional regulation, L–R interactions, and MMs across MDs, providing a mechanistic insight into the coordinated regulation during the MD processes.

### 3.6 Drug Target Prioritization for Metabolic Diseases

Identifying druggable targets is essential for translating L–R–TF regulatory networks into therapeutic strategies. Leveraging curated drug–target databases, we prioritized 63 key targets, comprising 23 receptors, 25 ligands, and 16 transcription factors. Notably, ICAM1 was classified both as a receptor and a ligand, indicating a dual role in bidirectional cell communication and highlighting its function as a central coordinator between immune and stromal cells in disease contexts.

**FIGURE 6:**
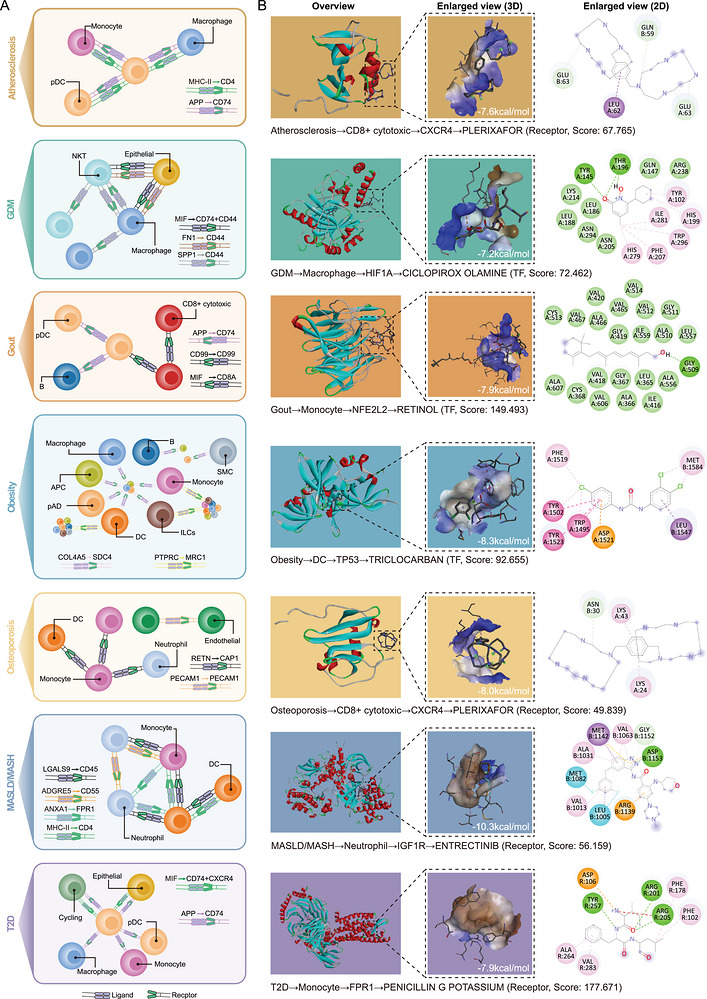
Molecular docking analysis of key drugs with each core target. (A) Key L–R pairs and their associated intercellular communication routes. (B) Molecular docking results between target drugs and core targets across metabolic diseases.

To evaluate therapeutic potential, we performed molecular docking analyses between top‐ranked candidate drugs and disease‐specific core targets (Figure 6B). In atherosclerosis, CXCR4, predominantly expressed in CD8^+^ cytotoxic T cells, displayed strong binding to plerixafor (drug score: 67.765; binding energy: −7.6 kcal mol^−1^). In GDM, the transcription factor HIF1A, enriched in macrophages, bound favorably to ciclopirox olamine (drug score: 72.462; binding energy: −7.2 kcal mol^−1^), suggesting potential modulation of hypoxia‐related signaling pathways. In gout, NFE2L2, highly expressed in monocytes, exhibited a robust interaction with retinol (drug score: 149.493; binding energy: −7.9 kcal mol^−1^), supporting its candidacy as a druggable transcription factor in monocyte‐mediated pathogenesis. For obesity, TP53 in dendritic cells (DCs) showed strong binding to triclocarban (drug score: 92.655; binding energy: −8.3 kcal mol^−1^), implicating TP53‐dependent pathways in DC‐linked immune dysregulation. In osteoporosis, CXCR4 again demonstrated significant interaction with plerixafor (drug score: 49.839; binding energy: −8.0 kcal mol^−1^), suggesting it may serve as a shared therapeutic target in CD8^+^ cytotoxic T cell populations across atherosclerosis and osteoporosis. In MASLD/MASH, IGF1R in neutrophils bound strongly to entrectinib (drug score: 56.159; binding energy: −10.3 kcal mol^−1^), representing one of the most stable interactions observed. Finally, in T2D, FPR1 in monocytes showed high‐affinity binding to penicillin G potassium (drug score: 177.671; binding energy: −7.9 kcal mol^−1^), highlighting its relevance as a druggable receptor in monocyte‐mediated disease mechanisms.

Together, these computational results demonstrate that core targets localized to disease‐relevant immune cell subsets exhibit strong binding affinities with existing compounds. This underscores that key regulatory mediators in metabolic diseases correspond to druggable nodes, providing promising therapeutic entry points to modulate cell‐type‐specific signaling, transcriptional regulation, and intercellular communication across diverse metabolic pathologies.

### 3.7 Interactive Web Tool for the Exploration of Metabolic Diseases

**FIGURE 7:**
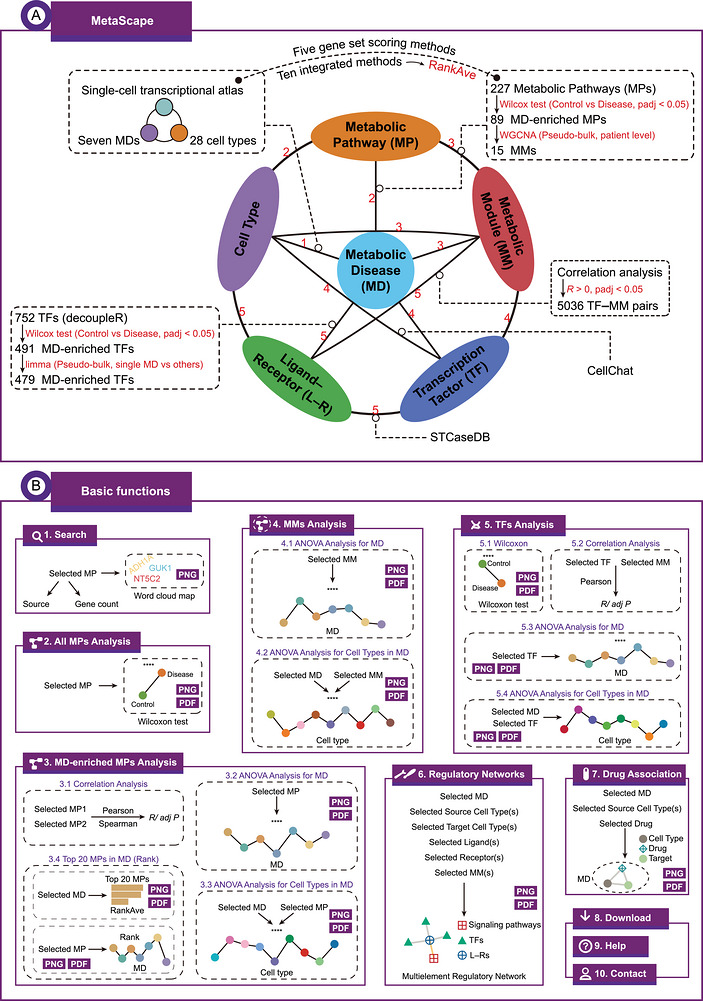
Functional overview of the Shiny tool. (A) Schematic of the core components of the Shiny platform. (B) Summary of major functions integrated within the tool.

A key objective of this study was to provide a comprehensive and accessible resource for investigating metabolic reprogramming in MDs. To this end, an interactive web tool was developed that integrates six core analytical elements and ten fundamental functions. The platform enables users to perform targeted analyses, explore L–R and TF networks, examine MM activity, investigate potential drugs and core targets, and compare findings with their own datasets (Figure 7A,B; see Methods). The tool is publicly available at https://scmetabolismexplorer.shinyapps.io/shiny/, offering an open‐access resource to facilitate in‐depth exploration in future metabolic research.

## 4 Discussion

Understanding how MPs coordinate to drive disease progression remains a central challenge in biomedical research. Here, we present a unified framework that systematically maps cooccurring MMs across diverse MDs at single‐cell resolution. The resulting metabolic atlas integrates large‐scale datasets to reveal organizational patterns of metabolic dysregulation that extend beyond individual pathways or cell types. While integrating systemic samples (e.g., PBMCs) with local microenvironment tissues (such as adipose tissue, placenta, and atherosclerotic plaques) inherently introduces biological heterogeneity, this cross‐tissue strategy is both deliberate and methodologically justified. Metabolic processes are highly dependent on the microenvironmental context [76]; thus, this heterogeneity reflects biologically meaningful variation, although it also represents an important interpretative consideration. Crucially, these diverse tissues share common cellular constituents, particularly circulating and tissue‐resident immune cells [77, 78, 79]. By leveraging robust algorithms like Harmony, which effectively aligns shared cell populations across batches while preserving tissue‐specific transcriptional features, we were able to seamlessly bridge systemic and local states. Consistent with recent pan‐tissue atlas efforts [80], this unified framework allows us to capture conserved principles of metabolic remodeling across diseases while maintaining the resolution to observe tissue‐specific metabolic adaptations.

Our findings indicate that MDs are associated with the coordinated activity of discrete modules, each governed by specific transcriptional programs and embedded within disease‐ and cell type‐specific networks. This systems‐level perspective provides new insights into disease heterogeneity, comorbidities, and underlying mechanisms. To quantify pathway activity at single‐cell resolution, we developed a specialized scoring framework and benchmarked multiple algorithms, identifying RankAve as the most reliable integrator. By combining complementary approaches, this strategy minimizes method‐specific biases and outperforms individual techniques in both single‐cell and bulk datasets. By applying this framework, we identified 89 MD‐related MPs and 15 coexpressed MMs, highlighting a modular organization of metabolic alterations rather than diffuse changes. For example, GDM showed widespread activation across multiple modules, reflecting global metabolic reprogramming. In contrast, T2D patients displayed either coordinated multimodule activity or dominance of a single module, providing a molecular explanation for the observed clinical heterogeneity. Specific modules, such as MM01 in MASLD/MASH and MM02 in gout, were selectively enriched, linking particular modules to lipid‐ or amino acid‐related disease mechanisms.

**FIGURE 8:**
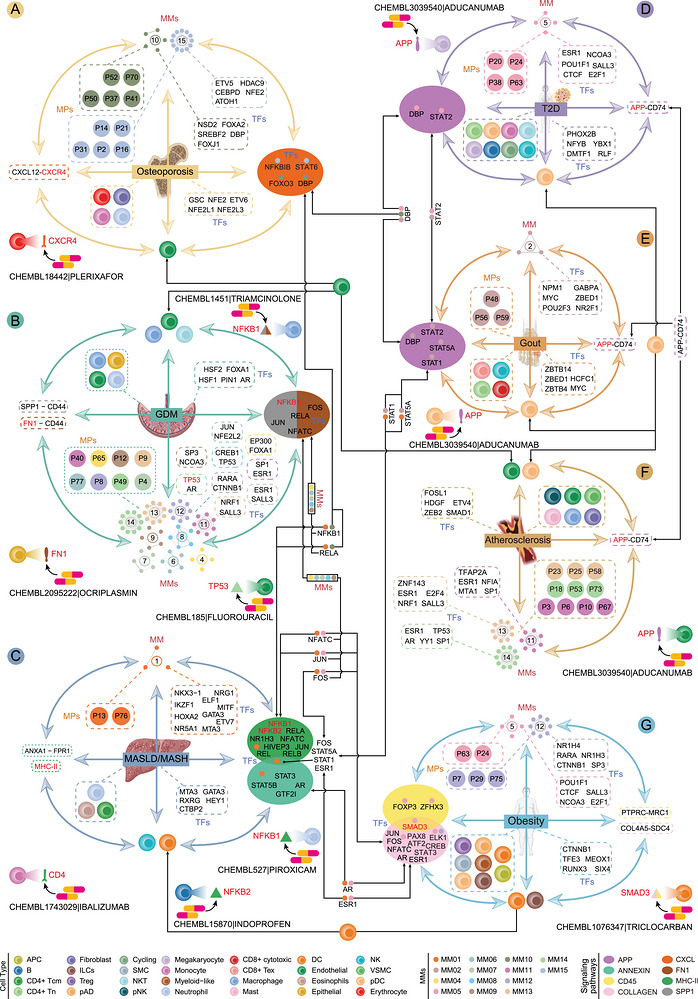
MD‐specific and shared complex mechanism network. (A–G) Key elements of regulatory networks in various MDs: (A) osteoporosis, (B) GDM, (C) MASLD/MASH, (D) T2D, (E) gout, (F) atherosclerosis, and (G) obesity.

Our analysis further revealed key TFs orchestrating these modules. STAT1 and STAT5A jointly regulate MM01 and MM02 through the MHC‐II, ANNEXIN, and APP signaling pathways, suggesting that immune‒metabolic crosstalk represents a core regulatory axis across diseases [81, 82]. Notably, the APP–CD74 axis, which emerges from interactions between plasmacytoid dendritic cells and macrophages, was consistently observed in T2D, gout, and atherosclerosis. Given its established role in promoting immunosuppressive responses, recurrent activation of this axis may allow chronic metabolic stress to persist while dampening local immune surveillance [83, 84]. This conserved interaction represents a potential unifying mechanism through which distinct MDs create an immunosuppressive microenvironment, facilitating disease progression. Importantly, several of these targets correspond to drug‐associated or potentially actionable candidates: APP is targeted by aducanumab in T2D, gout, and atherosclerosis (Figure 8D–F); the TF NFKB1 is linked to triamcinolone in GDM and piroxicam in MASLD/MASH (Figure 8B,C); SMAD3 is targeted by triclocarban (Figure 8G); and the receptor CXCR4 is the primary target of plerixafor in osteoporosis‐related signaling (Figure 8A).

Mechanistically, our observations indicate that a greater number of TFs associated with the regulation of metabolic dysfunction are shared between MASLD/MASH and obesity, with both the androgen receptor (AR) and estrogen receptor alpha (ESR1) present concurrently under these conditions (Figure 8C,G). This overlap suggests that lipid‐related metabolic disorders may converge on a common endocrine‒metabolic regulatory axis influenced by steroid hormone signaling. Given the well‐established roles of AR and ESR1 in lipid metabolism, inflammatory modulation, and crosstalk between hepatic and adipose tissues, their concurrent involvement implies that disruptions in hormone‐responsive transcriptional programs may underlie the transition from obesity‐induced metabolic stress to hepatic inflammation and fibrotic progression in MASLD/MASH [85, 86]. These shared TF signatures underscore a set of conserved regulatory circuits that could coordinate metabolic dysfunction across adipose and liver compartments, providing a mechanistic rationale for the strong clinical association between obesity and fatty liver pathology. Additionally, in the context of osteoporosis, we detected selective enrichment of the CXCL12–CXCR4 axis between endothelial cells and various myeloid cell populations (Figure 8A). This pattern suggests that CXCL12 derived from endothelial cells may actively recruit or retain CXCR4^+^ myeloid cells within the bone microenvironment, contributing to the inflammatory and remodeling imbalances characteristic of disease progression [87, 88]. Given the established role of the CXCL12–CXCR4 axis in regulating hematopoietic cell trafficking and mobilizing osteoclast precursors, its significant activation in this context points to a potential mechanism by which endothelial–immune crosstalk enhances osteoclastogenic activity and disrupts bone homeostasis. These findings imply that dysregulated endothelial signaling may serve as a critical driver linking immune cell dynamics to bone loss in osteoporosis.

Several limitations should be acknowledged. First, although this study conducted an integrated analysis of seven major MDs, expanding the cohort to include additional diseases would improve the generalizability of the findings. Second, these metabolic diseases are clinically and pathophysiologically interconnected, particularly in the setting of obesity and T2D; therefore, cross‐disease comparisons may partly reflect shared systemic dysregulation rather than entirely independent disease‐specific features. Third, the relationships among TFs, MMs, and L–R interactions were inferred computationally and require experimental validation in relevant in vitro or in vivo models. Fourth, the current analytical framework primarily captures transcriptional regulation based on available computational tools. However, metabolic states are influenced by multiple regulatory layers beyond gene expression and are highly context‐dependent. Accordingly, transcriptional regulators should be interpreted as indirect proxies of the underlying metabolic state. Future studies incorporating additional regulatory layers, such as epigenetic and post‐translational mechanisms, may further improve the characterization of metabolic dynamics. Fifth, because MMs were inferred from patient‐level pathway scores, module composition may exhibit moderate sensitivity to the choice of correlation metric. Although the overall module structure remained broadly consistent, the assignment of individual pathways—particularly those near module boundaries—may vary depending on the correlation method and should therefore be interpreted with caution.

In summary, our study represents an important step toward achieving a comprehensive understanding of the metabolic atlas for human MDs, laying a foundation for further investigations into module‐level functional coordination across diverse contexts. Moving forward, the research community will benefit from this open‐access metabolic atlas to help elucidate the biological mechanisms of diseases and accelerate the development of disease biomarkers and therapeutic targets.

## 5 Conclusions

In conclusion, this study presents a unified single‐cell metabolic atlas that reveals coordinated, module‐based metabolic dysregulation across diverse MDs. Our findings suggest that the transcriptional reprogramming of metabolism in these MDs is organized into statistically robust, co‐regulated MMs, rather than occurring as isolated or uncoordinated MP alterations. This coordinated transcriptional architecture provides a systems‐level explanation for disease heterogeneity and comorbidity. By integrating robust pathway‐scoring strategies, we identify key MMs, TFs, and immune–metabolic interaction axes with therapeutic relevance. This framework offers a valuable resource for advancing mechanistic understanding, guiding biomarker discovery, and generating hypothesis‐driven candidates for future targeted interventions in MDs.

## Author Contributions

**Y.P.Z**., **X.L**., and **Y.C**. contributed to conceptualization. **Y.P.Z**., **X.L**., and **Y.C**. designed experiments. **K.Y**., **C.X.H**., **H.Z**., **X.P**., **Y.M.Y**., **J.S.W**., and **D.P.M**. contributed to the data curation. **K.Y**., **C.X.H**., and **H.Z**. performed scRNA‐seq data analysis. **K.Y**., **C.X.H**., **H.Z**., **X.P**., and **Y.M.Y**. performed visualization and interpreted the results. **Y.P.Z**. provided resources. **K.Y**. and **X.L**. contributed to the software. **Y.P.Z**., **X.L**., and **Y.C**. supervised all work. **Y.P.Z**., **X.L**., and **C.X.H**. contributed to the funding acquisition. **Y.P.Z**., **K.Y**., **C.X.H**., **H.Z**., **X.P**., **Y.M.Y**., **J.S.W**., and **W.Q.J**. prepared the original manuscript. **Y.P.Z**., **X.L**., **Y.C**., **K.Y**., **C.X.H**., and **H.Z**. revised the manuscript. All coauthors read, reviewed, and approved the manuscript.

## Funding

This work was supported by grants from the National Science and Technology Major Program [2024ZD0530500], the National Natural Science Foundation of China [62472131, U23A20166, 32570792, 62502128, and 62172131], the Key Research and Development Program of Heilongjiang Province [2024ZX12C27], the China Postdoctoral Science Foundation [2024M760709], the Heilongjiang Postdoctoral Fund [LBH‐Z24210], and the Longjiang New Era Outstanding Doctoral Dissertation Project Grant [LJYXL2024‐069].

## Conflicts of Interest

The authors declare no conflicts of interest.

## Supporting information




**Supporting File 1**: advs76250‐sup‐0001‐SuppMat.docx.

**Supporting File 2**: advs76250‐sup‐0002‐TablesS1–S6.zip.

## Data Availability

The data that support the findings of this study are available in the Gene Expression Omnibus (GEO) database at https://www.ncbi.nlm.nih.gov/geo/, reference numbers GSE155960, GSE159677, GSE217561, GSE211783, GSE255566, GSE268210, GSE268211, GSE173193, GSE267033, GSE169396, GSE169397, GSE169398, GSE169399, GSE249311, GSE278204, GSE255075, GSE249997, GSE272133, GSE244120, GSE244118, GSE236746, GSE181674, GSE236610, GSE200744, GSE192587, GSE181646, GSE161042, and GSE145412. These data were derived from the following resources available in the public domain: GEO database, https://www.ncbi.nlm.nih.gov/geo/.
