# Supplementary material for: An Integrative Strategy Delineates Modular Metabolic Remodeling and Potential Therapeutic Targets Across Metabolic Diseases
Source: Adv Sci (Weinh). 2026 Jun 22:e76250. Online ahead of print. doi: 10.1002/advs.76250 (PMC13336389; doi:10.1002/advs.76250)
Supplement: Supplementary file 1 — Supporting File 1: advs76250‐sup‐0001‐SuppMat.docx. [file ADVS-9999-e76250-s002.docx]

**Supplementary Figures and figure legend**

**
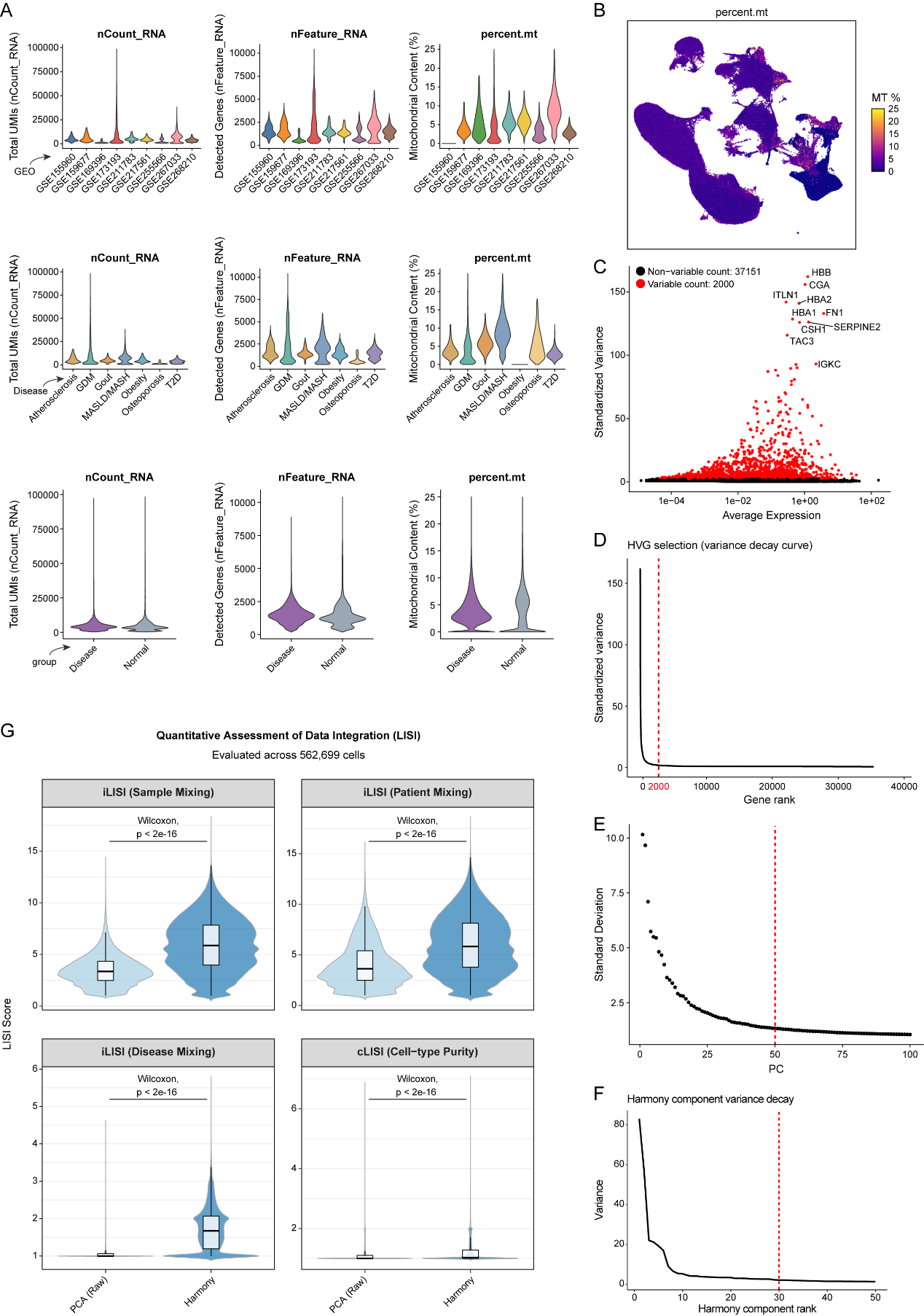
**

**Figure S1: Quality control, highly variable gene selection, and quantitative evaluation of multi-cohort data integration in single-cell transcriptomic data.** **A,** Violin plots displaying the distribution of quality control (QC) metrics across GEO datasets (top), different disease categories (middle), and disease vs control groups (bottom). **B,** UMAP projection of all cells colored by mitochondrial gene content (percent.mt). **C,** Scatter plot showing highly variable genes (HVGs) identified by Seurat. **D,** Variance decay curve for HVG selection. **E,** Elbow plot of principal component analysis (PCA), showing the standard deviation across the first 100 PCs. **F,** Variance decay curve of Harmony components after integration. **G,** Quantitative assessment of data integration using the Local Inverse Simpson's Index (LISI) evaluated across 562,699 cells before (PCA Raw) and after integration (Harmony). Violin plots with inner boxplots show integration LISI for sample mixing, patient mixing, and disease mixing, alongside cell-type LISI (cLISI) for cell-type purity. Two-tailed paired Wilcoxon tests.


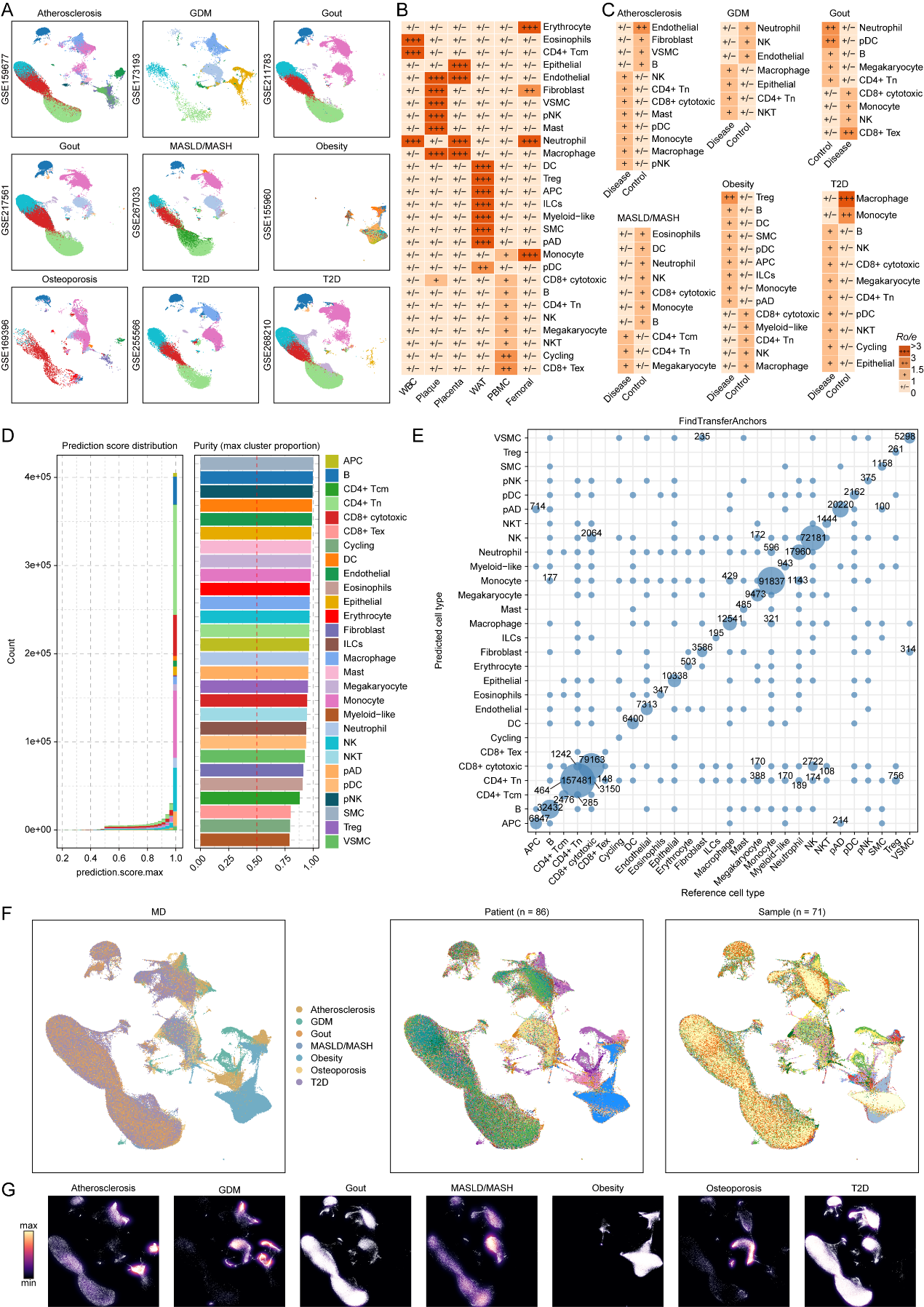


**Figure S2: The single-cell atlas of MDs.** **A,** UMAP plot showing the major cell types in each MD. Dots represent individual cells, and colours represent different cell populations. B, Overview of tissue preferences in MDs. **C,** Overview of the tissue preferences of various cell types in MDs. **D,** Prediction score distribution and purity of various cell types. **E,** Dot plot showing the reference-based label transfer strategy. **F,** UMAP plot showing the origins of the cells in terms of MD, patient, and sample. **G,** UMAP plot showing the cell density across MDs.


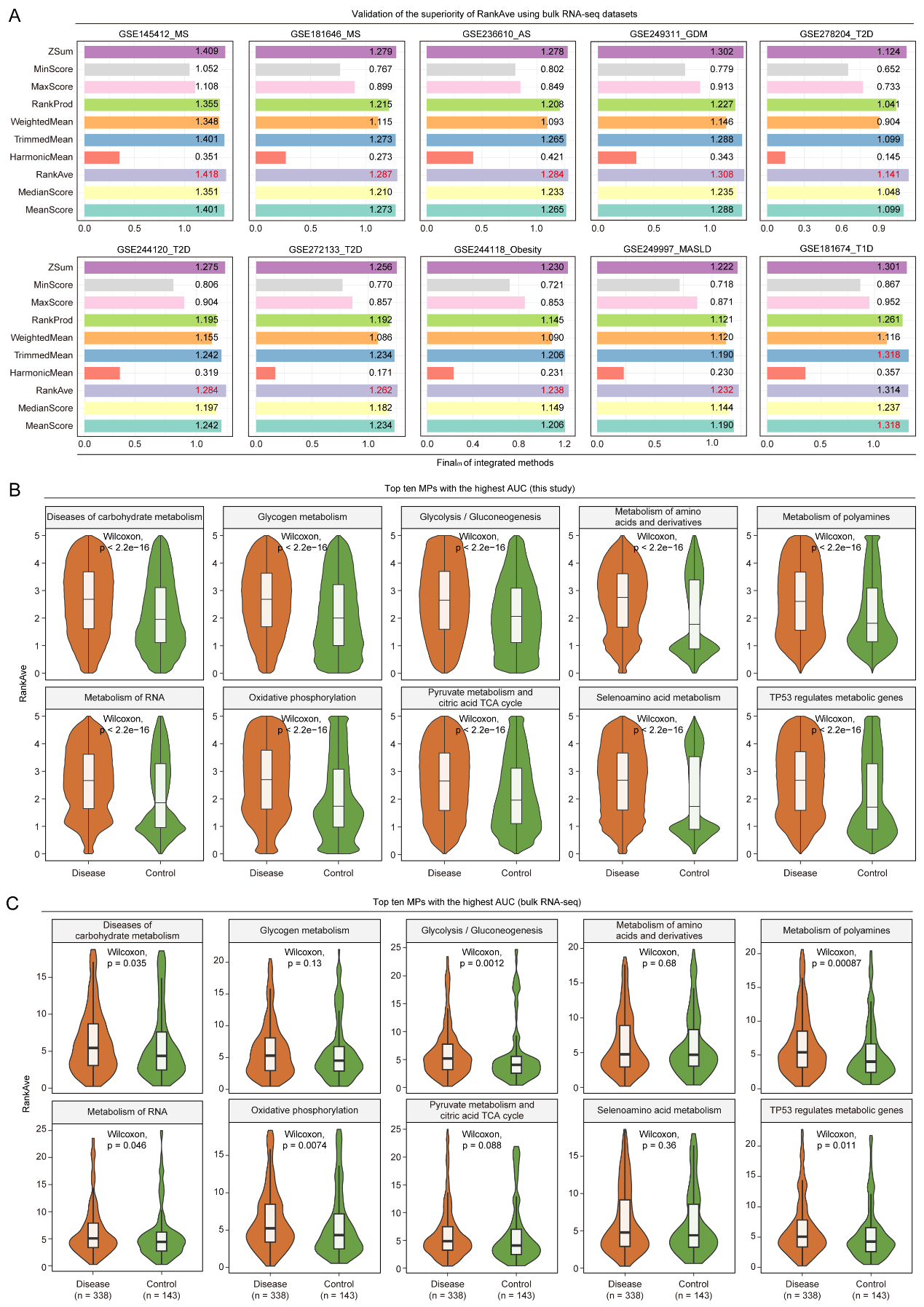


**Figure S3: Evaluation and validation of the metabolic scoring framework.** **A,** Validation of the RankAve method showing superior comprehensive performance in ten bulk RNA-seq datasets of MDs. **B,** The top ten MPs with the highest AUC in scRNA-seq data, showing RankAve score differences between disease and control groups. **C,** The top ten MPs with the highest AUC in bulk RNA-seq data, showing RankAve score differences between disease and control groups. Two-tailed paired Wilcoxon tests.


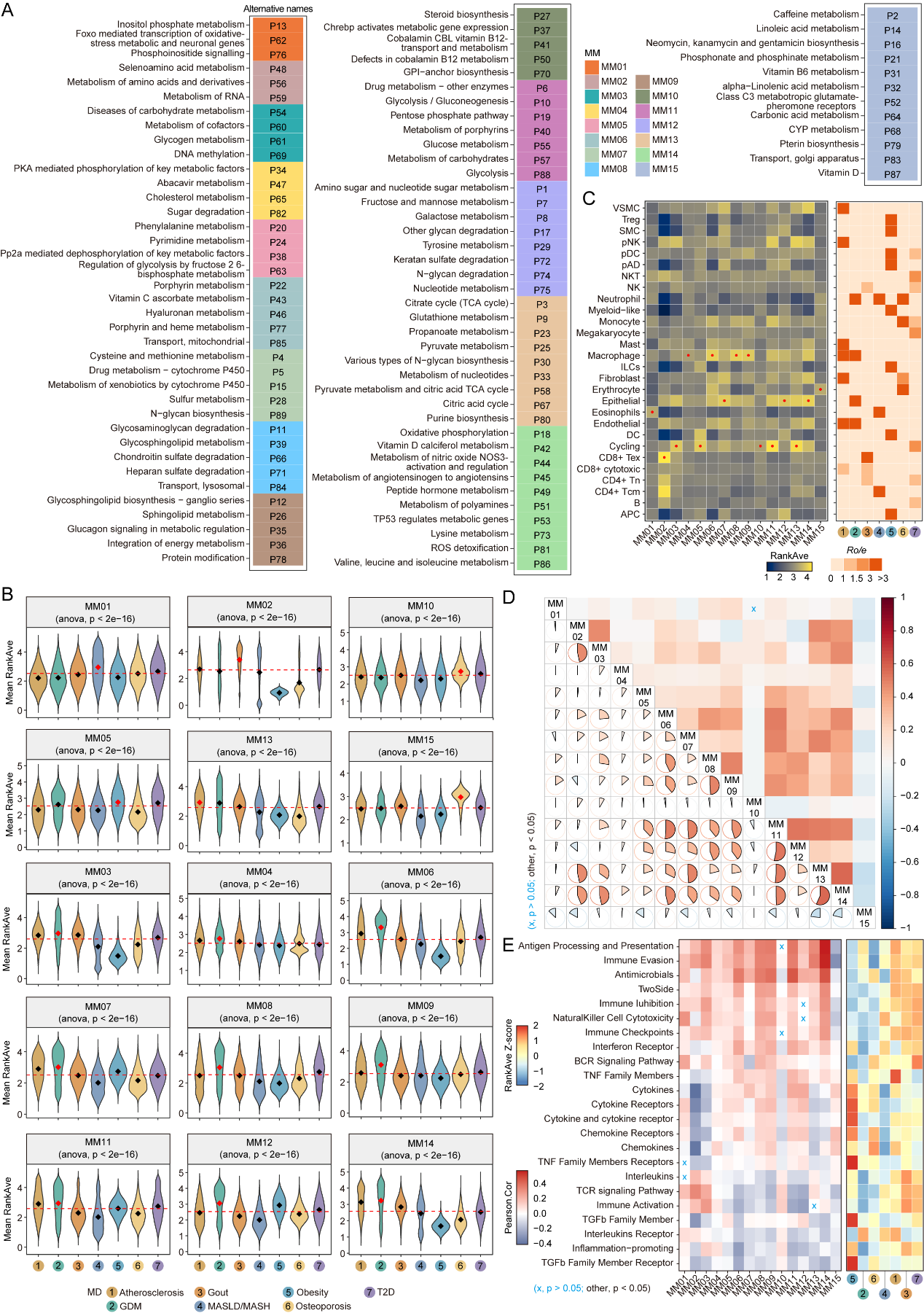


**Figure S4: Analysis of MM profiles and distribution.** **A,** Distribution of MPs across the identified MMs. **B,** Mean RankAve scores of MMs across MDs, with differences assessed using ANOVA. **C,** Heatmap of RankAve scores for MMs at the cellular level (left) and tissue preference of cell types across MDs (right). The red dots mark the cell types exhibiting the highest scores for each MM. **D,** Pearson correlation matrix of MM expression levels, presented as a fan plot (left) and as a heatmap (right), with non-significant correlations (*P* > 0.05) highlighted in blue. **E,** Heatmap depicting Pearson correlation between immune gene sets and MMs (left), and heatmap showing RankAve scores of immune gene sets across MDs (right).


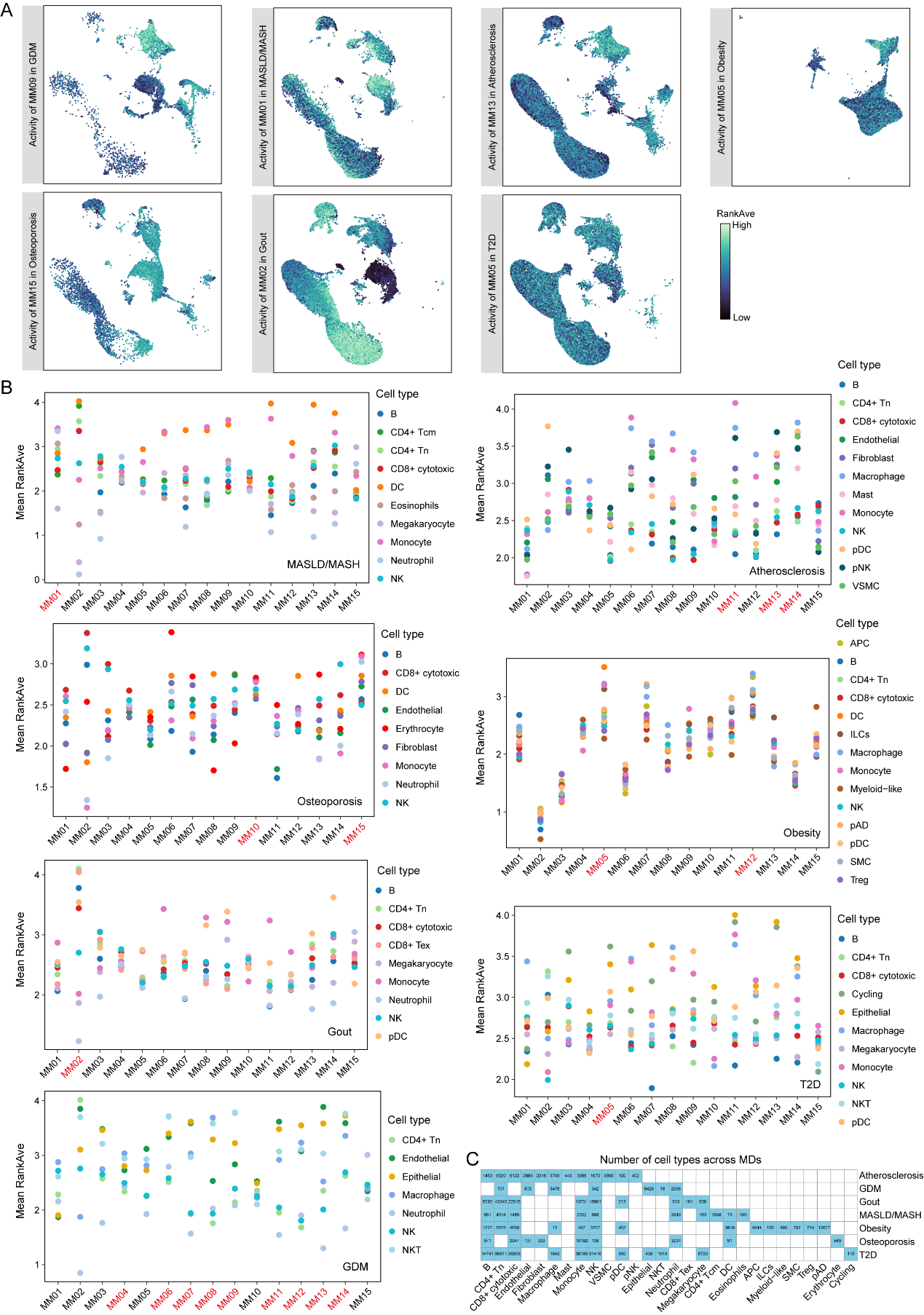


**Figure S5: Profiles and distribution of MMs.** **A**, UMAP plot showing the distribution of the most enriched MM in each MD, with colors indicating RankAve score levels. **B,** Scatter plot depicting the mean RankAve scores of MMs across MDs and cell types, with each point representing a distinct cell type. **C,** Distribution of cell types across MDs, with values indicating the corresponding cell counts.


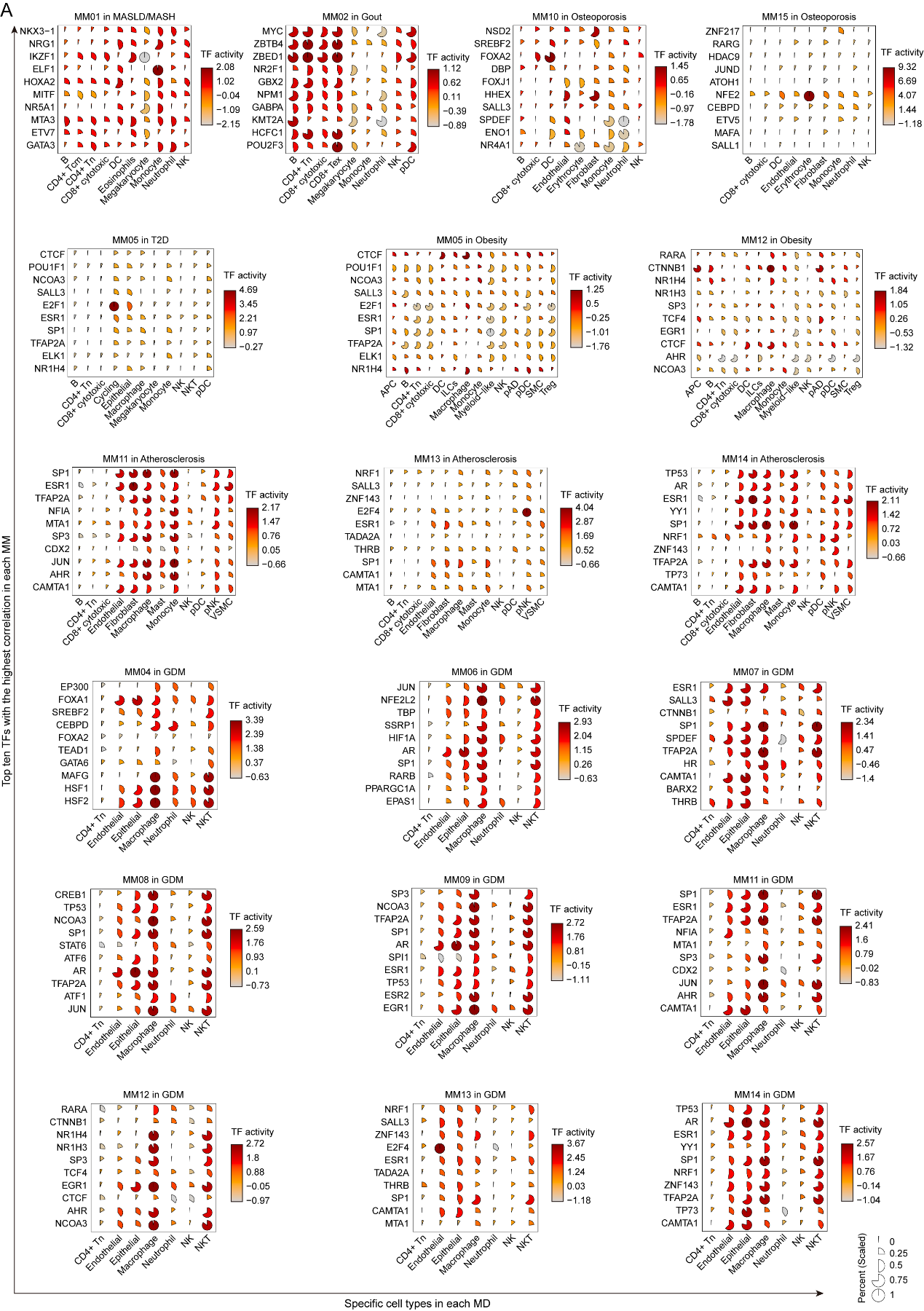


**Figure S6: Activity of the top ten TFs most strongly correlated with MMs across cell types in MDs. A,** Heatmap illustrating TF activity across distinct cell types involved in each MD. The color gradient reflects relative TF activity levels, while the size of each shape denotes the scaled percentage of TF activity.


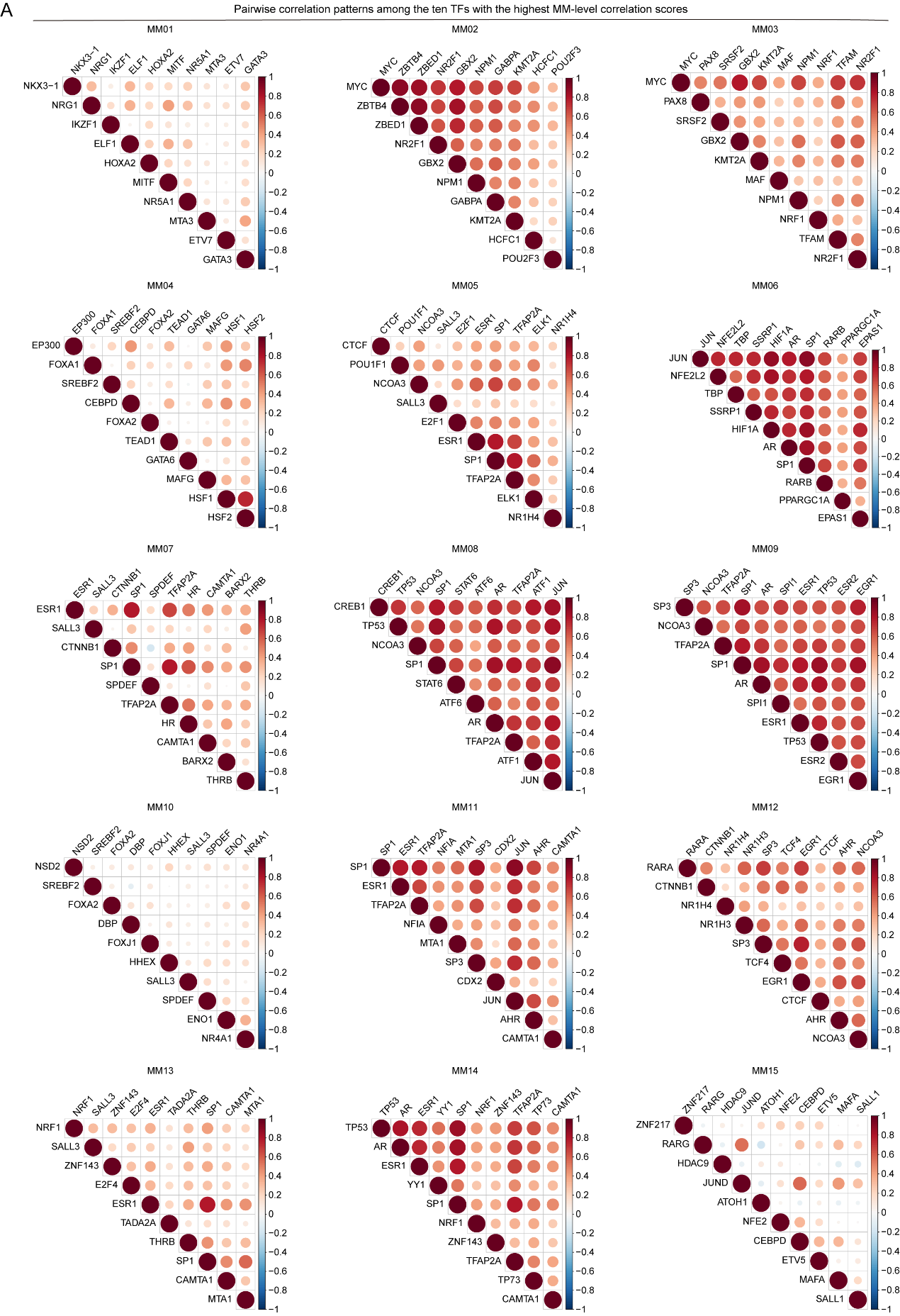


**Figure S7: Correlation heatmap of pairwise correlation coefficients of the top ten TFs in each MM. A,** Heatmap displaying the correlation coefficient matrix of the top ten TFs in each MM. The color gradient represents the magnitude of pairwise correlation coefficients: red corresponds to strong positive correlation (with the maximum value of 1), while blue corresponds to strong negative correlation (with the minimum value of -1). The color bar on the right panel indicates the quantitative correspondence between correlation coefficient values and the color scale.


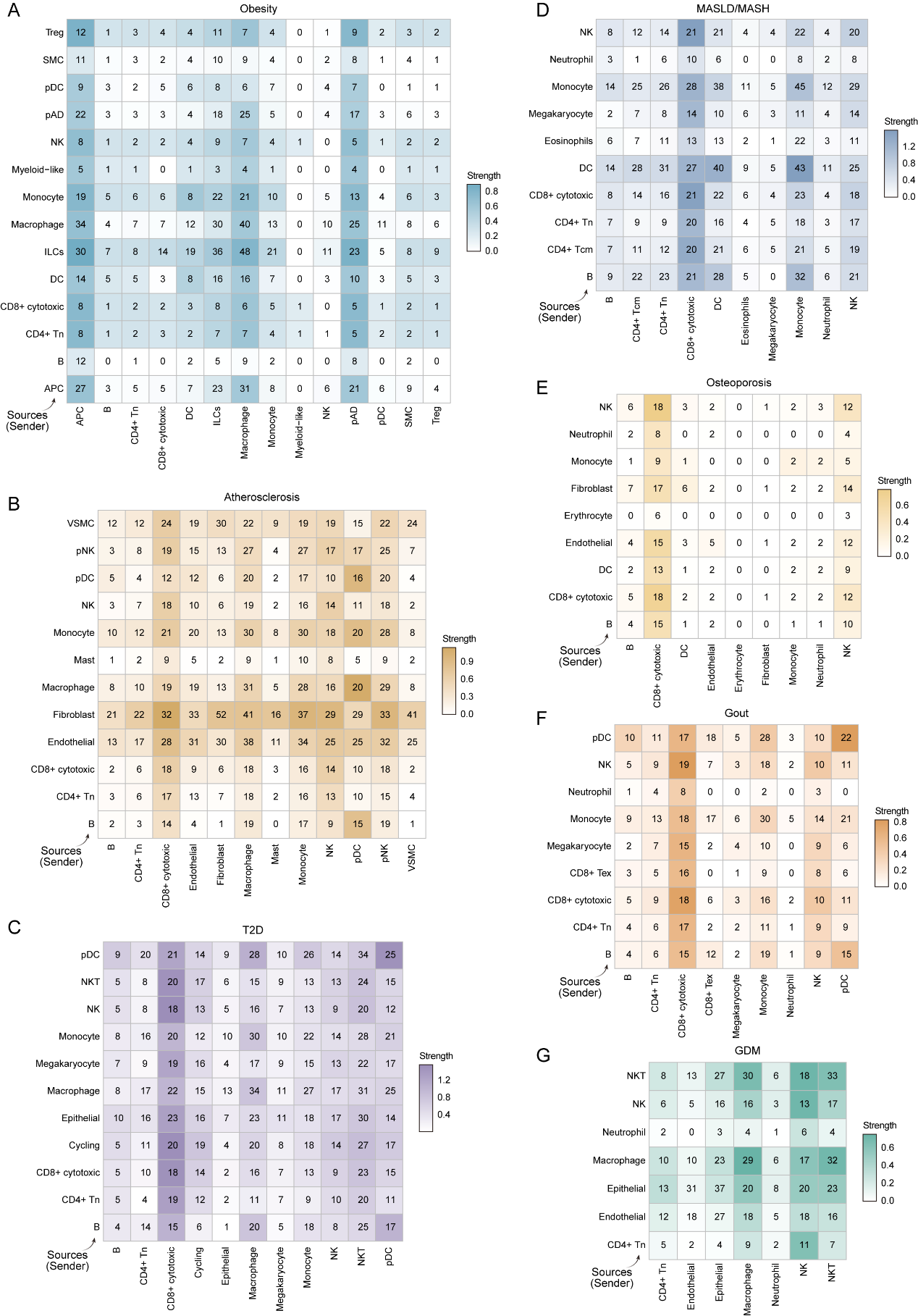


**Figure S8: Intercellular signal transduction in MDs.** **A**–**G,** Patterns of intercellular signaling among different cell types across MDs: (A) obesity, (B) atherosclerosis, (C) T2D, (D) MASLD/MASH, (E) osteoporosis, (F) gout, and (G) GDM. Numbers indicate the number of signals; color intensity reflects signal strength.


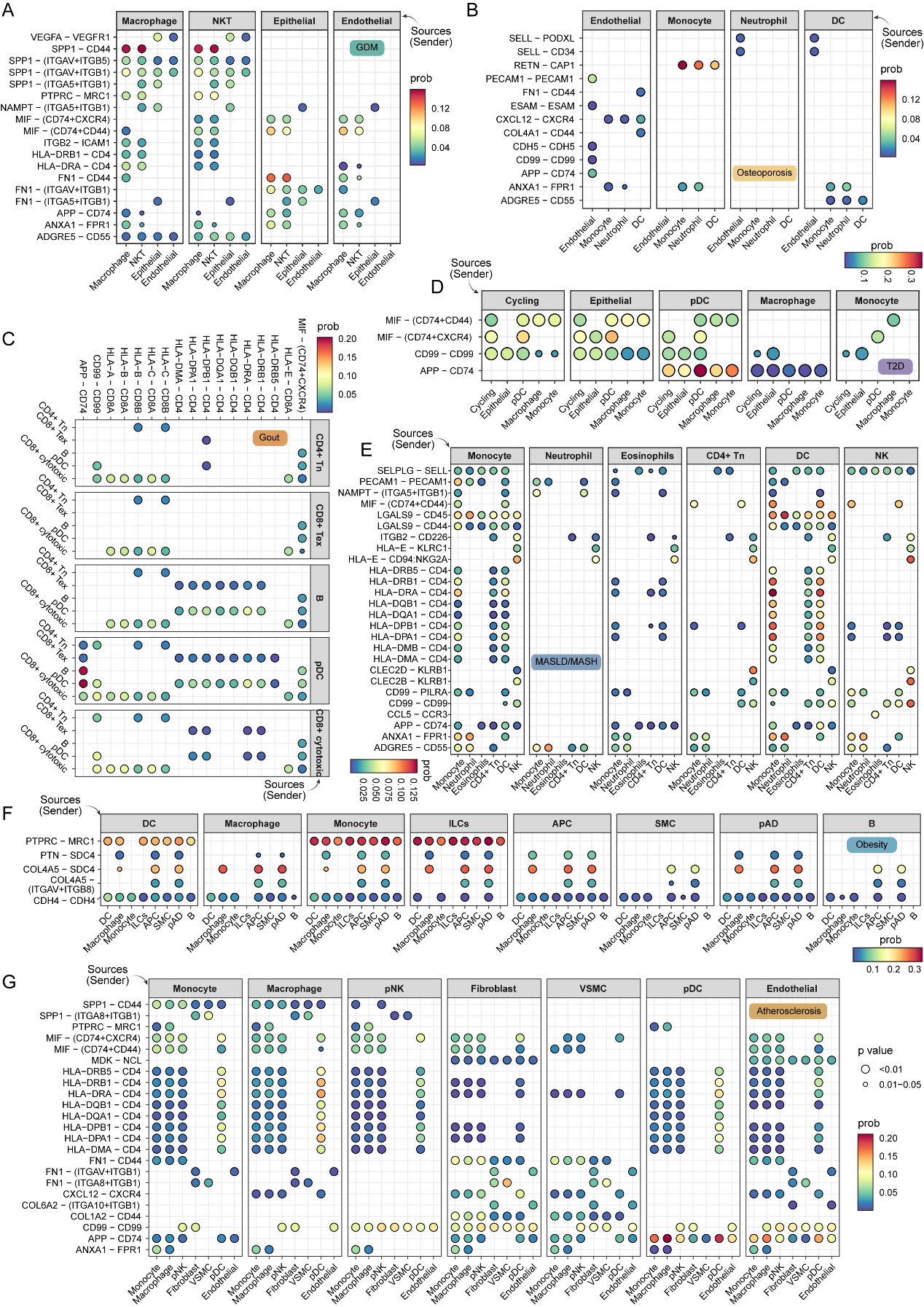


**Figure S9: Significant L**–**R pairs in MDs.** **A**–**G,** Dot plot illustrating significant L–R pairs among cell types in MDs: (A) GDM, (B) osteoporosis, (C) gout, (D) T2D, (E) MASLD/MASH, (F) obesity, and (G) atherosclerosis.

**Supplementary Table Information**

Table S1: Summary of datasets.

Table S2: Marker gene lists of cell types.

Table S3: Immune-related gene lists.

Table S4: Sources of metabolic pathways.

Table S5: Number of cell types across MDs, related to Figure S4C.

Table S6: Correspondence between Pearson- and Spearman-derived WGCNA MMs.
